# The hydrogenation side-reaction in copper-mediated radiofluorination

**DOI:** 10.1186/s41181-025-00384-1

**Published:** 2025-09-08

**Authors:** Sarandeep Kaur, Barbara Wenzel, Ramona Oehme, Claudia Wiesner, Klaus Kopka, Rareş-Petru Moldovan

**Affiliations:** 1 Department of Experimental Neurooncological Radiopharmacy, Helmholtz-Zentrum Dresden-Rossendorf (HZDR), Institute of Radiopharmaceutical Cancer Research, 04318 Leipzig, Germany; 2 Faculty of Chemistry and Food Chemistry, School of Science, Dresden University of Technology, 01069 Dresden, Germany; 3 Faculty of Chemistry, Leipzig University, 04103 Leipzig, Germany; 4 German Cancer Consortium (DKTK), Partner Site Dresden, 01307 Dresden, Germany; 5 National Center for Tumor Diseases (NCT), NCT/UCC Dresden, A Partnership Between DKFZ, Faculty of Medicine and Univ. Hosp. Carl Gustav Carus, TU Dresden & Helmholtz-Zentrum Dresden-Rossendorf (HZDR), 01307 Dresden, Germany

**Keywords:** Copper-mediated radiofluorination, CMRF, Fluorine-18, Hydrogenated side product, Protodeboronation, BEpin

## Abstract

**Background:**

Copper-mediated radiofluorination (CMRF) is a breakthrough in ^18^F-radiochemistry, enabling ^18^F incorporation into molecules even at electron-rich aromatic positions. In recent years, several improved protocols have been reported to advance the application of CMRF. These advancements primarily focus on improving radiochemical conversion, expanding substrate scope, and enabling scalability for remote-controlled radiotracer production. Despite these improvements, one major challenge remains: the protodemetallation. Protodemetallation is a common side reaction in transition metal-mediated cross-couplings that takes place by a mechanism that is not yet fully elucidated. In ^18^F-chemistry, the formation of the hydrogenated side product (HSP) can interfere with the chromatographic purification of the desired radiotracer, resulting in complex radiotracer production.

**Results:**

The present work investigates the factors influencing the rate of the hydrogenation reaction as well as the source of hydrogen in the CMRF by use of model precursors bearing -B(OH)_2_, -Bpin, -BEpin and -SnBu_3_ as leaving groups. While the CMRF reactions are usually carried out under anhydrous conditions, the formation rate of the HSP was evaluated by controlling the chemical constituents (type and molarity of reagents) as well as the physical parameters (time and temperature). Moreover, experiments with deuterated reagents complemented by high-resolution mass spectrometry (HRMS) analysis were carried out to identify the source of hydrogen for the reductive elimination step.

**Conclusion:**

This study identifies reaction parameters that influence hydrogenation side reactions in CMRF, enabling high RCC with minimal HSP formation. The optimal reaction conditions include low temperature, short reaction time, and minimal amount of precursor, copper, and ideally no base and alcohols as solvents. Among the evaluated precursors, –BEpin afforded the lowest HSP formation, while –B(OH)_2_ afforded the highest. Overall, this study showed that the selection of proper reaction reagents and the fine-tuning of reaction parameters can substantially reduce the HSP formation while maintaining optimal radiochemical conversion.

**Graphical abstract:**

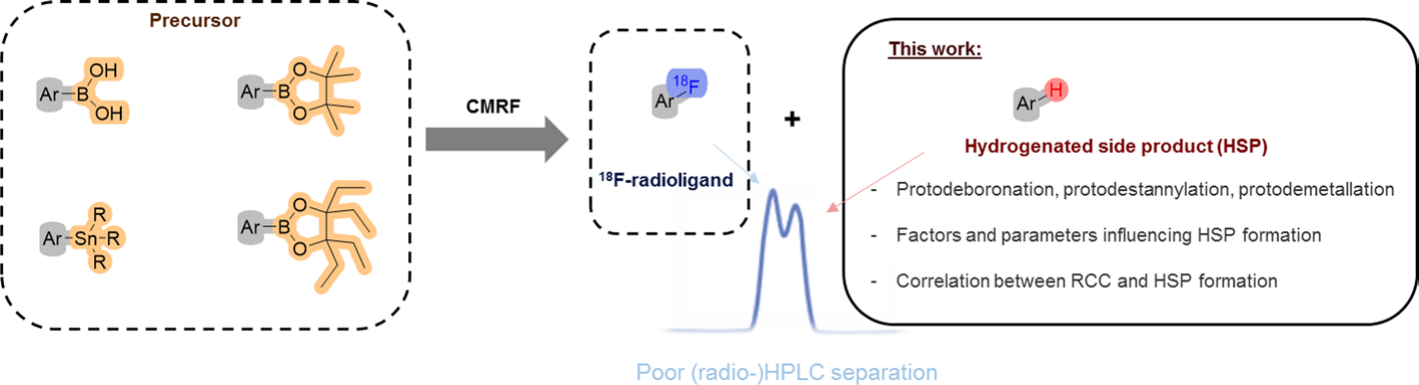

**Supplementary Information:**

The online version contains supplementary material available at 10.1186/s41181-025-00384-1.

## Background

**Scheme 1:**
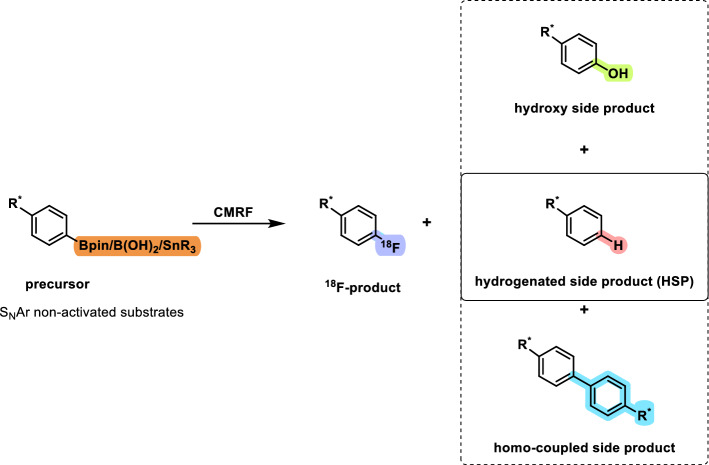
Copper-mediated radiofluorination (CMRF) and common side products

**Fig. 1:**
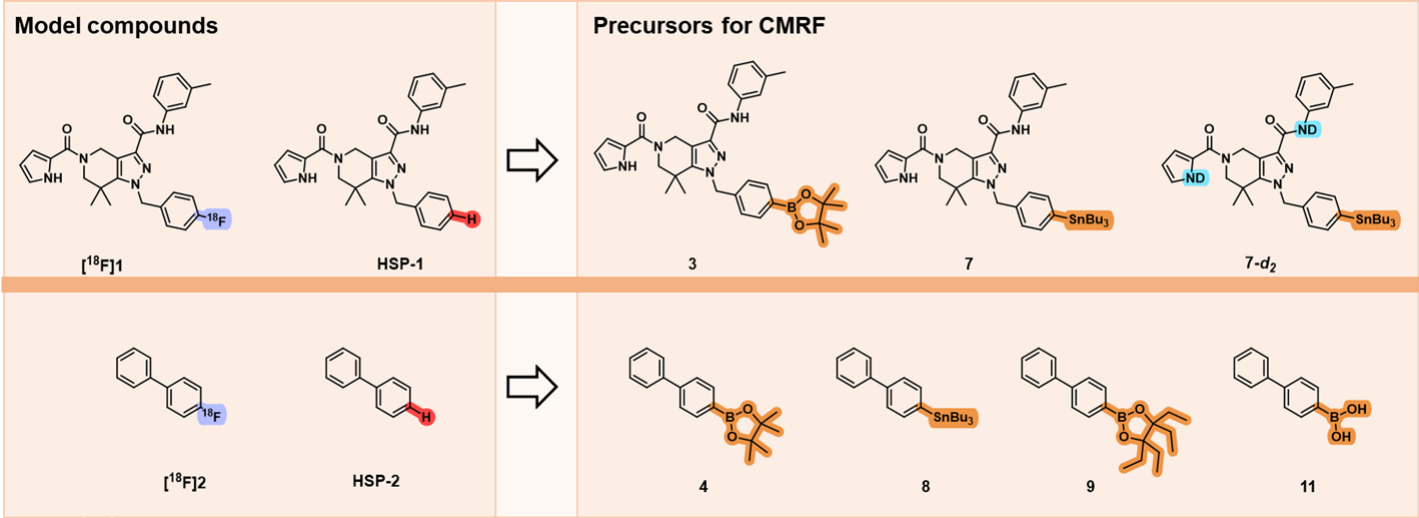
Overview of the structures of model compounds and precursors used in this study

Since the advent of positron emission tomography (PET) imaging, significant efforts have been made to advance the ^18^F-radiochemistry. Nevertheless, the development and application of novel radiofluorination methods that enable high radiochemical conversion (RCC) and radiochemical yield (RCY) with high molar activity (A_m_) continue to pose challenges for radiochemists, especially for molecules bearing electron-rich aromatic rings. Copper-mediated radiofluorination (CMRF) has made the greatest impact on ^18^F-radiochemistry in recent years. The origin of CMRF and improvements in its methodologies have been described in several recent reviews (Wright et al. 2020; Nandu et al. 2018; Mossine et al. 2016; Brooks et al. 2014; Deng et al. 2019; Preshlock et al. 2016; Bowden et al. 2025). Briefly, in 2013, Sanford et al. reported the Cu-mediated fluorination (CMF) on aryl boron reagents using an excess of KF and Cu(OTf)_2_ (Ye et al. 2013). The milder reaction conditions compared to the conventional fluorination methods for electron-rich aromatic rings and broader substrate scope with various functional group tolerances were investigated for the successful fluorination of aryltrifluoroborates (Ye et al. 2013). Thereafter, Gouverneur and co-workers successfully translated CMF ^18^F-radiochemistry and reported the first copper-mediated radiofluorination (CMRF) (Tredwell et al. 2014) using arylboronic acid pinacol esters (ArBPin) as precursors with [^18^F]KF/K222 and [Cu(OTf)_2_(Py)_4_] as mediators. This breakthrough in ^18^F-radiochemistry was found to be widely applicable due to its efficiency and ease of use for non-activated aromatic systems and was therefore followed by various protocol modifications and improvements. Later, Mossine and Makaravage et al. demonstrated that CMRF is not only effective for aryl- and vinylboronic acids but also for aryl- and vinylstannanes, expanding the applicability of this method beyond boronic esters to include a broader range of organometallic precursors (Mossine et al. 2015; Makaravage et al. 2016). Later, in 2017, they showed that customizing [^18^F]fluoride elution with tailored copper salt solutions can overcome base-sensitivity issues, significantly enhancing the compatibility, automation, and versatility of PET radiochemistry (Mossine et al. 2017). In 2015, Zlatopolskiy et al. (2015) introduced the “low base” and “minimalist” reaction conditions for CMRF to afford radiolabeled arenes with improved yields circumventing the need of time-intensive azeotropic drying step to prepare the ^18^F-reactant. In 2017, Zischler et al. further expanded this approach by discovering that primary and secondary alcohols significantly enhance CMRF, allowing minimalistic, base-free labeling of a wide range of pinacol and stannyl precursors (Makaravage et al. 2016; Zischler et al. 2017). Additionally, an efficient method for the preparation of the dry [^18^F]fluoride was developed for CMRF reactions. This approach utilizes quaternary ammonium, diaryliodonium, or triarylsulfonium salts in methanol to facilitate the release of [^18^F]fluoride trapped on an anion-exchange cartridge. By employing this technique, the need for the time-intensive azeotropic drying step is eliminated, streamlining the process and improving overall efficiency (Zischler et al. 2017). Moreover, this approach led to an improved RCC for various substrates (Zischler et al. 2017). Recently, Hoffmann et al. reported the evaluation of a series of Cu(II)-mediators for the radiofluorination of various model substrates bearing ‐B(OH)_2_ or ‐Bpin groups (Hoffmann et al. 2023). The use of these Cu(II) mediators and *n*-BuOH/DMI or DMI alone as solvents significantly improved the RCC, despite the use of lower amounts of precursors than conventionally employed (Hoffmann et al. 2023). Besides these achievements and ongoing developments in CMRF, the formation of the hydrogenated side product (HSP) has been largely unaddressed (Scheme 1). We believe the following factors contribute to this limited attention: first, not every CMRF results in HSP formation; it is generally considered substrate-specific, although the exact causes remain unclear. Second, many studies focus solely on optimizing RCC without pursuing further investigation into side products. Third, in the few cases where HSP formation has been reported, it was separated from the product via HPLC without examining its origin, as such analysis lies beyond the scope of those studies. Herein, hydrogenated side product (HSP) refers to the product of reactions commonly known as protodeboronation, protodestannylation (or generally referred as protodemetallation) and hydrodehalogenation. The HSP is not the only by-product in CMRF, hydroxy and homo-coupled side products might also be formed (Scheme 1). Due to their different chromatographic properties, the OH- and the homo-coupled side products can be usually well separated from the radiotracer by semi-preparative HPLC. However, the HSP has a similar polarity compared to the fluorinated product, which can lead to difficulties in the HPLC separation process. It often requires time-consuming extensive optimization of the HPLC conditions and might result in long retention time or the requirement of special stationary phases such as pentafluorophenyl (PFP) or mobile phases such as tetrahydrofuran (Lai et al. 2021; Mossine et al. 2018; Kaur et al. 2025; Bernard-Gauthier et al. 2018). For example, we recently reported the radiosynthesis of **[**^**18**^**F]1** (Fig. 1) for targeting mutated isocitrate dehydrogenase (mIDH), which required an HPLC purification step with a retention time (*t*_R_) of around 73 min to successfully eliminate the **HSP-1** (Kaur et al. 2025). Moreover, this is an important aspect, because the HSP might have a similar binding affinity to the biological target of interest as the radiotracer and therefore influence the imaging and the accuracy of molar activity determination, specifically the "effective molar activity" which considers UV by-products as defined in the literature (Lai et al. 2021; Kaur et al. 2025; Coenen et al. 2017). Hence, to ensure reliable outcomes, careful examination of by-product formation and a rigorous purification of the final product are imperative.

In general, HSP formation is a known challenge of transition metal-mediated reactions like for the Cham-Lam (Cu-mediated) and Suzuki/Stille (Pd-mediated) coupling reactions. Despite the prevalence of these transition metal-mediated reactions, the precise mechanism by which the organic residue is transferred from boron to the aromatic ring via the transition metal remains unclear (Thomas et al. 2018). The same applies to the formation of the by-products that occur during the cross-coupling reactions. In the absence of a metal catalyst, the protodeboronation of aryl boronic acids takes place in aqueous solution and is pH-dependent, being accelerated under basic conditions (Cox et al. 2016, 2017). In general, it is proposed that the formation of highly reactive aryl boronate anions ([ArB(OH)_3_]^–^) takes place, followed by *ipso*-protonation concerted with C-B cleavage (Hayes et al. 2021). Several methods to diminish or suppress the formation of the undesired HSP include the use of anhydrous solvents, the use of masked boronic acids (e.g., boronic esters, trifluoroboronates), or specially designed catalysts (Hardouin Duparc et al. 2018; Chen et al. 2020). While a number of studies investigate the formation of the HSP in organic chemistry, the ^18^F-radiochemistry literature is still sparse in this regard (Lai et al. 2021; Mossine et al. 2018; Stenhagen et al. 2013; Sun et al. 2024). While the use of anhydrous reaction conditions is widely employed in ^18^F-radiochemistry, the use of masked boronic acids and specially designed transition metal reagents has only marginally been investigated in the CMRF (Wright et al. 2020). Moreover, other reaction conditions that might influence the formation of HSP in the CMRF, like temperature, stoichiometry, type of base, additives (e.g. alcohol), etc., have hitherto not been investigated. Thus, the present study aims to evaluate the reaction parameters that might influence the formation of the HSP in the “classical” CMRF using [Cu(OTf)_2_(Py)_4_] as a mediator. In general, the rate of formation of the HSP in various reaction conditions was assessed by HPLC-analysis of aliquots of the reaction mixture. Deuterated experiments were carried out in order to identify the source of hydrogen responsible for the HSP formation and the rate of deuterium incorporation was determined by mass spectrometry.

## Results

For the present study, the tetrahydropyrazolopyridine-based compound **[**^**18**^**F]1** and the structurally less complex fluorobiphenyl **[**^**18**^**F]2** have been selected as model compounds to systematically investigate the formation of the respective hydrogenated side products **HSP-1** and **HSP-2** (Fig. 1). For the radiofluorination reactions, the corresponding aryl-boronic acids (–B(OH)_2_), boronic acid pinacol esters (–Bpin), boronic acid tetraethylpinacol ester (–BEpin), (Hadjipaschalis et al. 2025; Craig et al. 2025) and tributylstannanes (–SnBu_3_) (Fig. 1) were used as precursors and [Cu(OTf)_2_(Py)_4_] as copper mediator. Whereas most of the CMRF tests were performed using the biphenyl compounds, experiments with deuterated reagents for the mechanistic study were performed exclusively on the tetrahydropyrazolopyridine compounds due to their ionizability and thus, their suitability for mass spectrometry analysis to determine the rate of deuterium incorporation. The synthesis of precursors and reference compounds is described in detail in Supporting Information S1.

In the Suzuki–Miyaura type cross-coupling, the HSP is usually formed during the reaction by the water-mediated pinacol boronic ester hydrolysis followed by the formation of highly reactive arylboronate anions, and it has been shown that HSP formation is greatly reduced under anhydrous conditions (Lennox and Lloyd-Jones 2010). The CMRF reactions are usually carried out in anhydrous solvents, and therefore, we initially assumed that the HSP formation is either taking place by the aqueous quenching of the reaction or during the reaction in case alcohol is used as an additive to enhance the radiofluorination. Additionally, other chemical and physical factors might influence the rate of formation of the HSP and therefore, the study was designed in order to investigate the influence of *(i) aqueous quenching of the reaction mixture; (ii) alcohol as co-solvent; (iii) different phase-transfer catalysts (PTCs); (iv) reaction time; (v) different leaving groups of precursor; (vi) amount of precursor; (vii) amount of base (K*_*2*_*CO*_*3*_*); (viii) acidic protons of precursor; (ix) reaction temperature; and (x) molar amount of copper-complex.* As a prerequisite for the study, the radiolabeling conditions to radiosynthesize **[**^**18**^**F]1** and **[**^**18**^**F]2** with high RCC from respective -Bpin precursors **3** and **4** were optimized (Details in Supporting Information S2) and the HPLC separation of the hydrogenated side-products from their respective (radio)fluorinated products **1** and **2** were established (Supporting Information S3). A pentafluorophenyl (PFP)-modified column demonstrated superior resolution for both compound classes (Details in Supporting Information S3).

**Scheme 2:**
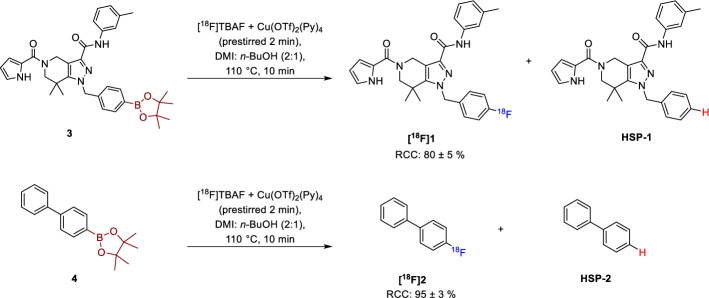
CMRF of **[**^**18**^**F]1** and **[**^**18**^**F]2** with the formation of respective protodeboronated side products (**HSP-1** and **HSP-2**)

In brief, the azeotropically dried [^18^F]fluoride and [Cu(OTf)_2_(Py)_4_] in DMI were pre-stirred at r.t. for 2 min, followed by the addition of respective precursor (**3** or **4**) in *n*-BuOH, and the resulting reaction mixture was stirred at 110 °C for 10 min (Scheme 2). This standard procedure was then stepwise modified according to the investigated parameters. The RCC was routinely assessed by radio-TLC and periodically analyzed using radio-HPLC. For quantification of the HSP, aliquots of the cooled reaction mixture were analyzed using isocratic HPLC conditions. The amount of **HSP-1** and **HSP-2** was calculated from the respective calibration curves of the reference compounds determined under the same HPLC conditions. Additionally, in the case of experiments with deuterated reagents, the reaction mixture was subjected to solid-phase extraction (SPE), followed by semi preparative HPLC separation to isolate **HSP-1**. The fraction containing **HSP-1** was concentrated under reduced pressure and the product was analyzed by high-resolution mass spectrometry (HRMS) to determine the extent of deuterium incorporation. All experiments were performed at least in duplicate.

### Aqueous quenching of reaction mixture

While the CMRFs are carried out in anhydrous solvents, the short reaction time and substoichiometric use of [^18^F]fluoride compared to classical transition metal-catalyzed cross-couplings imply that a large amount of unreacted precursor is still be present in the reaction mixture at the end of the reaction. The HSP can be formed either during the labeling reaction due to trace amounts of water and/or during the aqueous quenching step. To investigate whether the HSP formation takes place during the aqueous quenching step, **[**^**18**^**F]1** was synthesized according to the standard procedure mentioned above in DMI alone, and the cooled reaction mixture (RCC ~ 60%) was divided into two parts. One half was quenched with H_2_O and the other one with D_2_O and both solutions were subjected to SPE followed by HPLC separation to isolate the hydrogenated side product and to determine the rate of deuterium incorporation. Contrary to our expectations, HRMS analysis of the isolated side product from the reaction quenched with D_2_O revealed only **HSP-1**, with no evidence of the deuterated side product (**DSP-1**) and was comparable to the exclusively formed **HSP-1** in the reaction quenched with H_2_O. In conclusion, the aqueous quenching of the reaction did not contribute to the HSP formation in CMRF of **[**^**18**^**F]1**. This indicated that the HSP formation takes place during the reaction, possibly due to the influence of other parameters or even due to the presence of trace amounts of moisture. Sources of moisture can include the reaction solvents, reaction vessel, the atmospheric humidity for the manually performed experiments, or a trace amount of water that remains solvated with the dry [^18^F]fluoride reagent. While the glass, round bottom reaction vessel (glass, round bottom) was oven-dried before use, the commercially purchased solvents used for our reactions contain ≤ 0.045% H_2_O (~ 0.023 µmol), which might lead to the formation of up to 0.046 µmol of HSP. In the manually performed reactions, air moisture might play a marginal role although the reactions are sealed after adding the reagents.

### Alcohol as co-solvent

The alcohol-enhanced CMRF was firstly reported in 2017 by Zischler et al. (2017) resulting in an enormous impact on ^18^F-radiochemistry. The use of alcohols such as MeOH or *n*-BuOH as co-solvents eliminated the time-consuming azeotropic drying and increased the RCC (Zischler et al. 2017). Furthermore, this method allowed practical “last-stage” access to ^18^F-fluorinated indoles, phenols, and anilines from unprotected precursors, demonstrated through the preparation of 6-[^18^F]fluorodopamine (6-[^18^F]FDA) and 6-[^18^F]-L-fluoro-L-3, 4-dihydroxyphenylalanine (6-[^18^F]FDOPA) (Zischler et al. 2017). However, large amounts of precursor (60 μmol) were needed to achieve high RCC, which is neither ideal nor practical for the preparation of ^18^F-radiopharmaceuticals. Later, Zhao et al. (2022) studied alcohol-enhanced CMRF using a variety of model reactions in which the preparation of dry [^18^F]fluoride and the amount of precursor, base, and [Cu(OTf)_2_(Py)_4_] was varied. As a result, the authors found that the addition of a base enabled the use of reduced precursor quantities (e.g., 15 μmol) and adequate levels of [Cu(OTf)_2_(Py)_4_] (6.6 µmol) without compromising RCCs (Zhou et al. 2022). Although the introduction of alcohols as co-solvents in CMRF was an important improvement in terms of RCC, their effect on the HSP formation as a source of acidic protons has not been investigated to date.

To evaluate the role of alcohol as co-solvent, **[**^**18**^**F]1** was synthesized by the standard procedure using three different solvent conditions: (i) DMI, (ii) DMI:*n*-BuOH 2:1, and (iii) DMI:*n*-BuOD-*d*_*10*_ 2:1. For the evaluation of **HSP-1** formation, the reaction mixtures with DMI and DMI/*n*-BuOH were quenched with H_2_O and the mixture with DMI/*n*-BuOD-*d*_*10*_ with D_2_O. Then, the solutions were subjected to SPE followed by semi-preparative HPLC purification to isolate the hydrogenated products and to determine the amount of **HSP-1** and the rate of deuterium incorporation using HRMS.

**Fig. 2:**
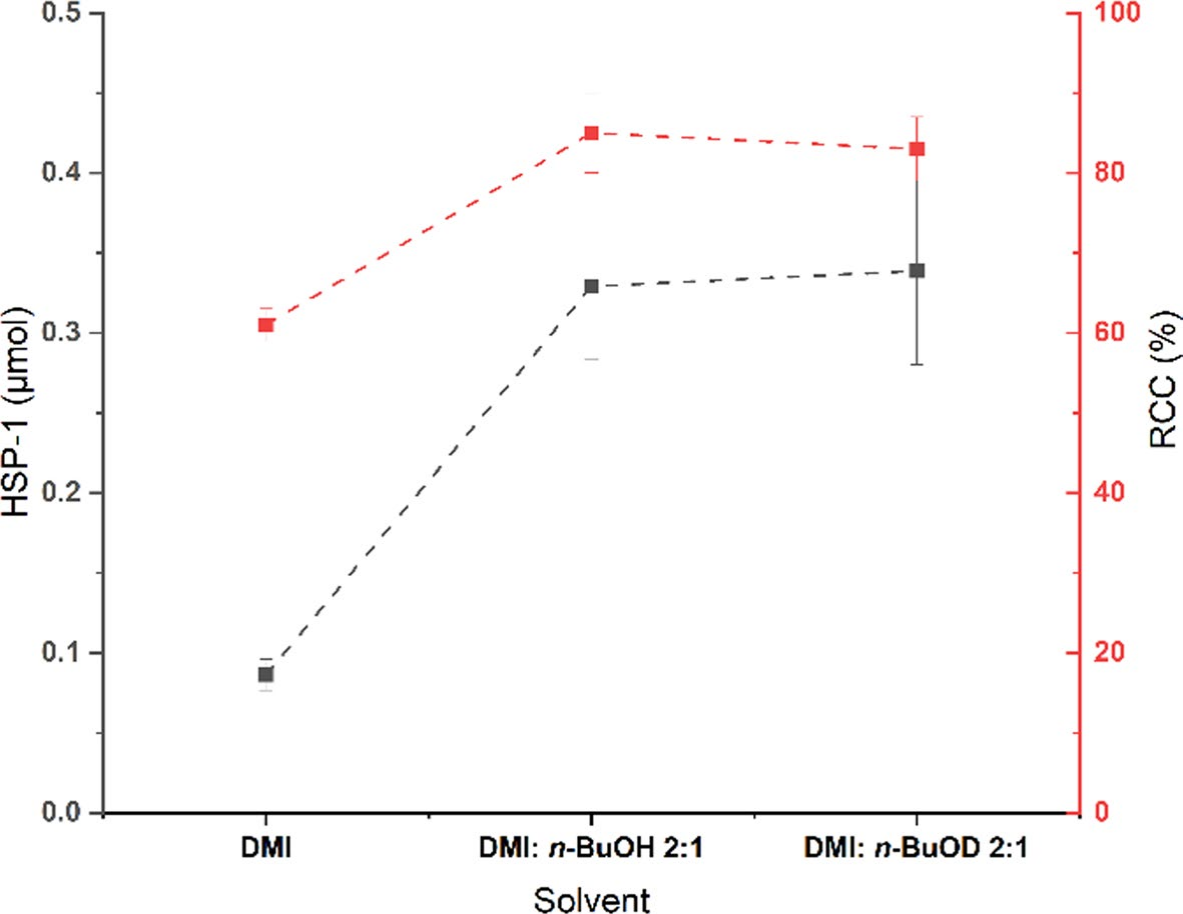
Effect of alcohol (*n*-BuOH/*n*-BuOD-*d*_*10*_) as co-solvent on the **HSP-1** formation during the CMRF of **[**^**18**^**F]1**

The RCC of **[**^**18**^**F]1** determined for the reactions carried out with alcohol as co-solvent (DMI: BuOD-*d*_*10*_ or BuOH 2:1) was about ~ 85%, whereas the reaction with DMI alone had an RCC of 61% and thus, in agreement with the findings reported by Zischler et al. (2017) regarding the effect of alcohol on the CMRF. However, the addition of alcohol as a co-solvent increased the **HSP-1** formation by four-fold in comparison to the reaction done in DMI alone (Fig. 2) reflecting a general faster reaction kinetics. The discrepancy between the increased RCC and the yield of HSP formation can be explained by the molarity of the reaction: while the [^18^F]fluoride is almost completely consumed after 10 min, large amounts of protodeboronatable precursor are still present in the reaction mixture, suggesting that strict control of the reaction time may be necessary to keep HSP formation low while achieving an optimal RCC. Notably, the HRMS quantification of the isolated HSP from the reaction performed with BuOD-*d*_*10*_ revealed only a trace of deuterium incorporation (Supporting Information S7), which suggests that other sources contribute significantly to the formation of **HSP-1** and the increased rate of hydrogenation is related to the faster reaction kinetics rather than the influence of alcohol as source of protons.

In organic chemistry, the mechanism of HSP formation was investigated in various transition metal coupling reactions. In 1978, an early mechanism for catalytic hydrodehalogenation of aryl halides was proposed by Zask et al. (1978) and then further extended by Nolan et al. (Navarro et al. 2004) involving oxidative addition of aryl halide (Ar–X) to a Pd(0) complex, followed by displacement of the halide ligand by methoxide, β-elimination of formaldehyde and reductive elimination of Ar–H to regenerate the Pd(0) species leading via a four-step mechanism (Supporting Information S5). In 2013, Ahmadi et al. performed a mechanistic investigation of the role of alcohol in the HSP formation in the Pd-catalyzed cross-coupling reactions Supporting Information S5) (Ahmadi and McIndoe 2013). The oxidation of the alcohol (solvent) was observed on the cationic Pd-complex and the kinetic isotopic investigations revealed that the deprotonation of the alcohol on the cationic palladium complex was the key step in the mechanism of the HSP formation (Supporting Information S5) (Ahmadi and McIndoe 2013). However, most prior mechanistic studies have focused on Pd-catalysts and, therefore, the underlying mechanisms between the two metals may vary. Nevertheless, a detailed mechanistic and kinetic investigation is necessary to confirm the role of alcohol deprotonation in HSP formation during CMRF, as the Cu-complex is not used in catalytic amounts and the radiofluorination reactions are usually short time (10–15 min), suggesting that the mechanism may slightly differ.

### Different phase transfer catalysts and reaction time

Phase transfer catalysts (PTCs) like Kryptofix 2.2.2 or quaternary ammonium salts are typically used for radiofluorination (Wright et al. 2020). Herein, the effect of different PTCs (TBAHCO_3_, TEAHCO_3_, TBAOTf) and reaction time on the RCC and **HSP-2** formation was investigated for the CMRF of **[**^**18**^**F]2**.

**Fig. 3:**
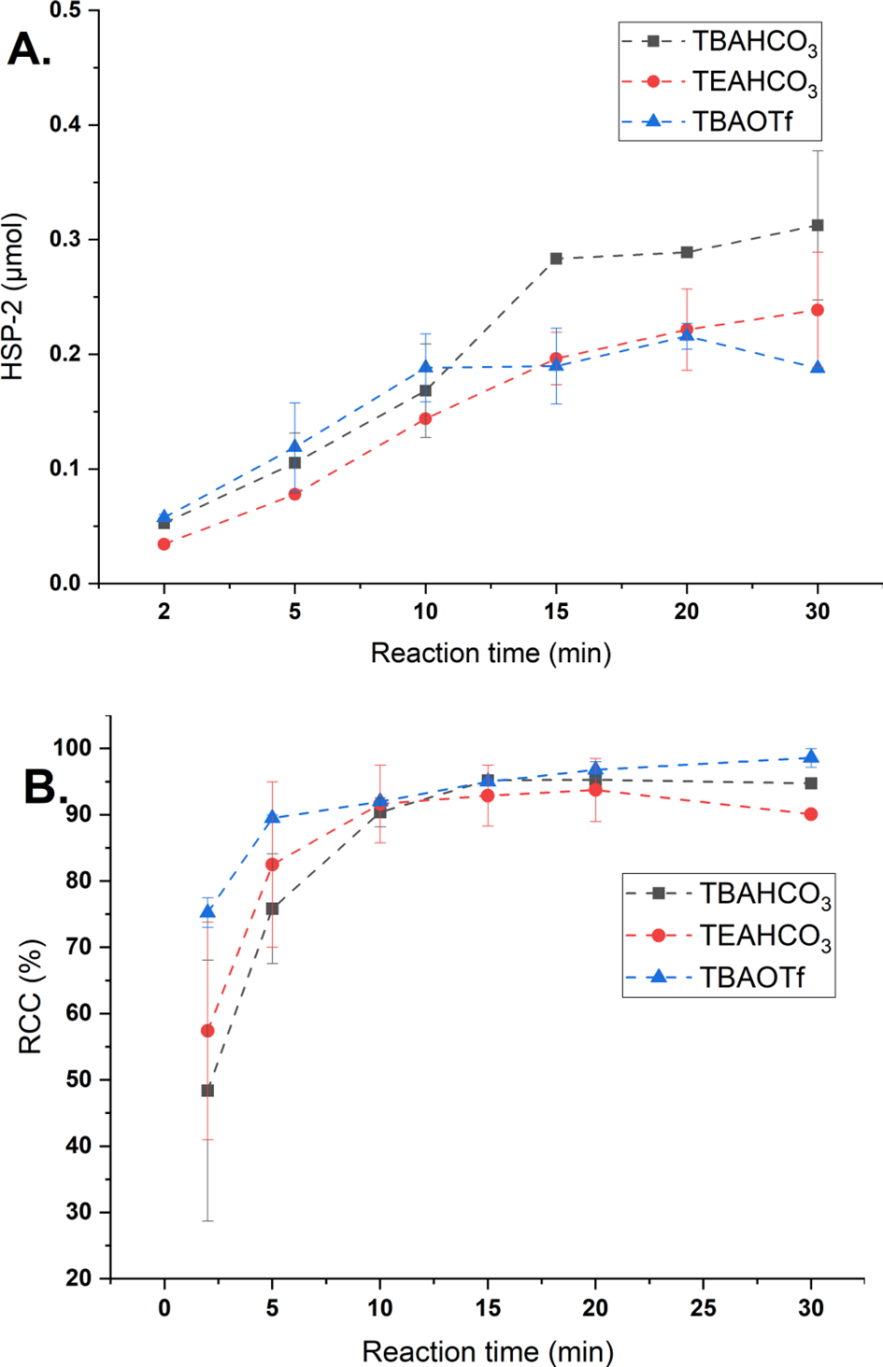
Time-dependent influence of different phase transfer catalysts on the **HSP-2** formation (**A**) and RCC (**B**) of the CMRF of **[**^**18**^**F]2**

The results showed that there was not a major difference in the **HSP-2** formation with these different CMRF PTCs (Fig. 3). As general trend, the HSP formation is constantly increasing with the reaction time until about 15 min followed by a slower reaction progress or stagnancy. In the first 10 min, the reaction with TEAHCO_3_ appears to result in slightly lower **HSP-2** formation as compared to the reactions with TBAHCO_3_ and TBAOTf. In contrast to the HSP formation, the RCC of **[**^**18**^**F]2** increased within the first 5–10 min of reaction and afterwards reached a stagnancy independent of the PTC used (Fig. 3B), underlining the different stoichiometry of the two reactions as detailed in the previous section. The use of TBAOTf as PTC outperformed TBAHCO_3_ and TEAHCO_3_ in terms of optimum RCC (> 70%) and lowest **HSP-2** formation (0.05 µmol) at 2 min of reaction. Overall, this study indicates that the reaction time plays an important role and it should be kept as short as possible to avoid the formation of large amounts of HSP at acceptable RCC.

### Different leaving groups of precursor

The choice of precursor plays an important role in the radiofluorination reactions and, especially, in terms of RCC. The most common types of leaving groups used in CMRF are boronic esters (-B(OR)_2_), boronic acids (-B(OH)_2_), and alkylstannanes (-SnR_3_). The pinacol esters (-Bpins) are widely used nowadays as they can usually be synthesized starting from commercially available building blocks, and they often result in higher RCC as compared to the -B(OH)_2_ and -SnR_3_ (Wright et al. 2020; Mossine et al. 2015; Makaravage et al. 2016). The -SnR_3_ precursors are generally toxic, whereas -B(OH)_2_ precursors are sometimes difficult to handle and purify. Although the use of -Bpin precursors has resulted in highly promising methods in CMRF, they have their limitations as well. The -Bpin precursors are usually unstable and prone to hydrolysis to B(OH)_2_, therefore resulting in purification challenges which can sometimes lead to fluctuating yields in the radiofluorinations (Oka et al. 2022). Recently, the development and investigation of -BEpins was reported for the Pd-catalyzed Suzuki − Miyaura couplings in organic chemistry (Oka et al. 2022). The authors showed largely improved stability and straightforward purification of -BEpin vs. -Bpin precursors via silica gel column chromatography. The improved precursor stability was also reflected in the yield of the cross-coupling reaction, with the -BEpins giving much higher yields than the -Bpins, most likely due to the reduced amount of side reactions (Oka et al. 2022). The newly reported -BEpins have been evaluated in the present study, for the CMRF of **[**^**18**^**F]2** Fig. 1) for the **HSP-2** formation, alongside -B(OH)_2_, and -BPin analogs. During the revision process for this publication, the use of BEpins in the CMRF was reported simultaneously by Hadjipaschalis et al. (2025) and by Craig et al. (2025) The 4-biphenyl BEpin precursor **9** was efficiently synthesized through the dehydrative esterification of the 4-biphenyl boronic acid **11** (Supporting Information S1), without major loss on the silica gel column and stability challenges, consistent with the previous report on –BEpins (Oka et al. 2022).

Although the study initially aimed to investigate the four precursors **4**, **8**, **9**, and **11**, the tin precursor **8** was excluded due to its degradation under the experimental conditions (as detailed below). As an alternative, the influence of the tin-bearing precursor was subsequently evaluated using **7** in comparison to the Bpin-bearing precursor **3** on **HSP**-1 formation.

**Fig. 4:**
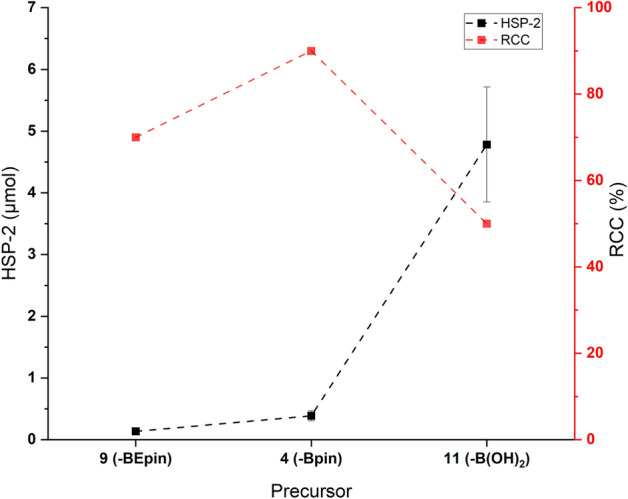
Effect of precursor type for CMRF of **[**^**18**^**F]2** on the **HSP-2** formation and RCC

The evaluation of the three different boron-based precursors (**4**, **9** and **11**) in the CMRF of **[**^**18**^**F]2** revealed that the use of the boronic acid precursor **11** resulted in the formation of considerably higher amounts of **HSP-2** as compared to the -Bpin (**4**) and -BEpin (**9**) after 10 min of reaction time under standard conditions (Fig. 4). The lowest amount of **HSP-2** was obtained with the use of the -BEpin precursor. However, under the given reaction conditions this precursor gave a slightly lower RCC (70%) in comparison to the -Bpin (**4**) (90%). In contrast, recent literature reports have shown higher or similar RCC values with -BEpin precursors compared to -Bpin precursors (Hadjipaschalis et al. 2025; Craig et al. 2025). This deviation likely stems from differences in the reaction protocols used. Our preliminary studies to improve the RCC with -BEpin precursor** 9** showed that using DMI/*n*-BuOH (2/1, *v/v*) instead of DMI alone improved the RCC from 70 to 94%, but at the cost of ~ 3 -fold higher **HSP-2** formation, likely due to increased reaction kinetics. Further optimization of reaction conditions, such as temperature, solvent, phase-transfer catalyst and copper mediator might enhance the RCC of CMRF using -BEpins, however, this was not part of the present study. Thus, careful optimization of reaction conditions is critical to improve RCC while keeping the HSP formation low.

Mossine et al. also reported increased side-product formation with -B(OH)_2_ precursors during the CMRF of ^1^⁸F-labeled 4-acetophenone (Mossine et al. 2015, 2018). The significant formation of **HSP-2** in the case of the boronic acid precursor **11** could be caused by the acidic proton(s) of the precursor during the transmetallation step, and the fast transfer of H to the arene (Thomas et al. 2018). The auto/self-condensation property of boronic acids to boroxines and the water formed as a result of the auto-trimerization of boronic acids might also play a role in the increased formation of HSP (Supporting Information S5) (Turco 2021; Clair et al. 2014; Liang et al. 2023; Noonan and Leach 2015). Moreover, the presence of base accelerates the auto-condensation of the boronic acids (Turco 2021; Clair et al. 2014; Liang et al. 2023) which might be a hint that also in the CMRF the base might play a role in the HSP formation. However, further investigation is required to confirm the assumption that boroxine formation takes place during the CMRF. Intermolecular hydrogen bonding in boronic acids as neat reagents can also lead to protodeboronation as reported by Noonan et al. (2015). It was found that the reduction in the entropy of activation in the solid state of boronic acids promotes protodeboronation (Noonan and Leach 2015). It was also observed that little protodeboronation occurs in the presence of moisture, suggesting that water alone is not the causative agent in protodeboronation of neat boronic acid samples and a solid-state pre-organization effect promoted by the presence of the required supramolecular interactions seems to be more likely (Noonan and Leach 2015). It was experimentally supported by the fact that little or no decomposition was observed for notoriously unstable boronic acids, e.g. 2-furanylboronic acid when stored in solution for prolonged periods as compared to the neat solid-state storage (Noonan and Leach 2015). Thus, it is not excluded that the protodeboronation starts before CMRF, however, the HPLC investigation of neat **11** revealed no **HSP-2** in the solid state. Nevertheless, it cannot be excluded that the entropy of activation required for supramolecular arrangements for protodeboronation can also be reached under the reaction conditions applied for radiofluorination.

As an alternative to boron-based precursors, the use of tin-precursor **8** (-SnBu_3_, Fig. 1) was attempted. However, precursor **8** was unstable to moisture degrading to biphenyl (**HSP-2**) during HPLC analysis/purification prior to CMRF (Supporting Information S4), thus complicating the quantification of the **HSP-2** formed during the CMRF of **[**^**18**^**F]2** *vs.* that resulting from the degradation of precursor **8** itself. Its instability also explains the low RCC obtained with precursor **8** (Supporting Information S4). Nevertheless, this underlines the importance of considering precursor stability when designing and optimizing radiolabeling reactions (Yuan et al. 2020). However, SnBu_3_-based precursors are widely applied in CMRF and therefore it is of interest to evaluate the formation of HSP by using stable precursors. Thus, in the present study, the impact of -SnBu_3_ bearing precursor **7** (Fig. 1) *vs.* -Bpin bearing precursor **3** on the **HSP-1** formation was evaluated by the CMRF of **[**^**18**^**F]1**. Our results revealed that the use of precursor **7** led to a 21% lower formation of **HSP-1** compared to the reaction with precursor **3** (Supporting Information S4). However, the lower **HSP-1** formation with **7** was accompanied by a reduced RCC (21% *vs.* 60% for **3**), likely due to the use of general reaction conditions in this study, which are not specifically optimized for the tin-precursor **7**, which is a limitation of the present study.

Overall, given the lowest HSP formation and optimal RCC, the -BEpin precursor **9** presents a better alternative in comparison to the corresponding -Bpin, -B(OH)_2_ and -SnBu_3_ precursors. To generally assess the scope and limitations of using -BEpin precursors in CMRF, further studies are needed.

### Amount of precursor

**Fig. 5:**
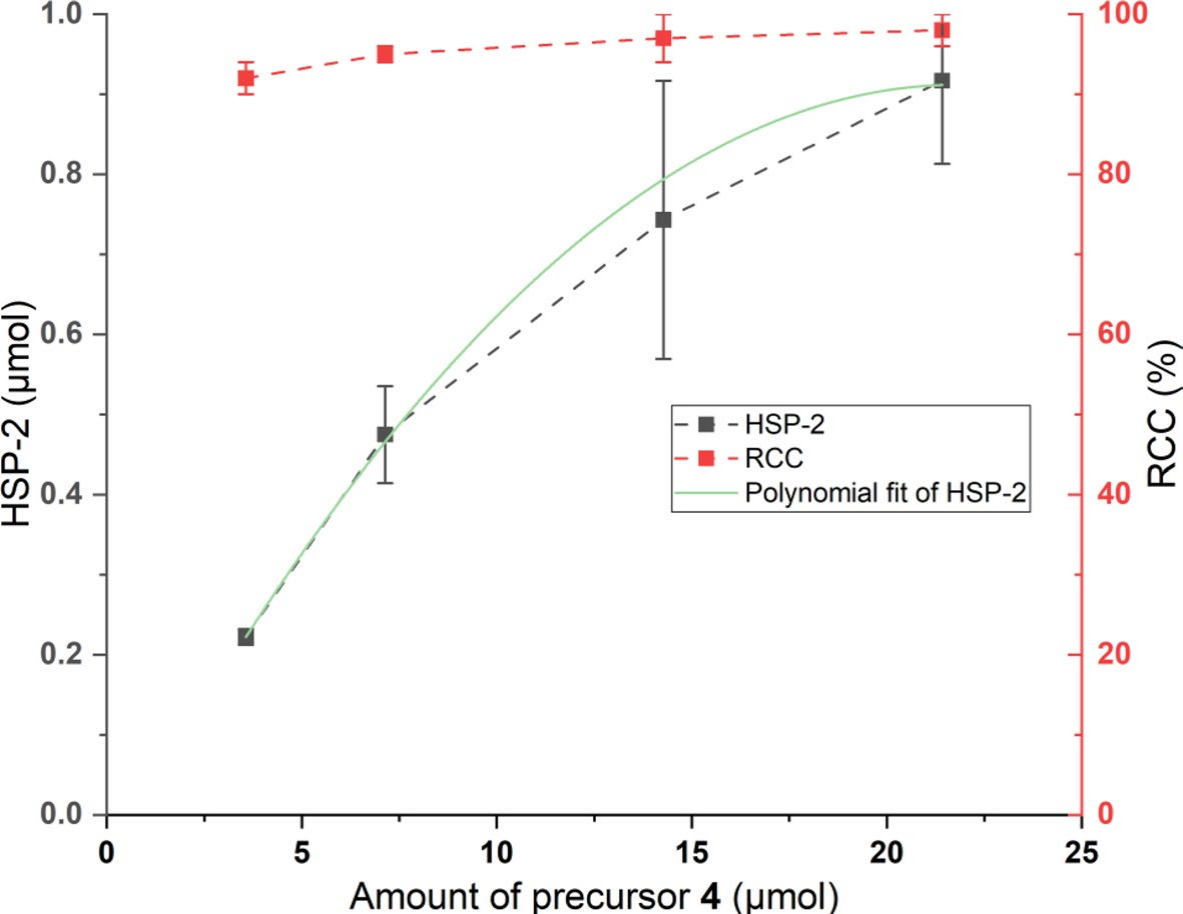
Effect of amount of precursor **4** (3.6, 7.1, 14.3 and 21.4 µmol) on the formation of **HSP-2** and RCC in CMRF of **[**^**18**^**F]2**

The amount of precursor used in the CMRF is one of the most important reaction parameters, usually optimized to achieve high RCC by using as low amount of precursor as possible. The effect of the amount of precursor on the **HSP-2** formation was investigated by using 3.6, 7.1, 14.3 and 21.4 µmol of the -Bpin precursor **4**. As shown in Fig. 5, there was almost no difference observed for the RCC ranging from 92 to 98%. However, the impact on the formation of **HSP-2** was considerable (Fig. 5). The **HSP-2** formation increased with the increase of precursor amount and can be best described as a polynomial curve as shown in Fig. 5. As already discussed, -Bpin precursors are often unstable and prone to hydrolysis, resulting in the formation of the corresponding -B(OH)_2_ analogs. Thus, higher quantities of precursor **4** may lead to elevated trace levels of boronic acid **11**, which could significantly enhance HSP formation, and explain the effect shown in Fig. 5. In addition, due to the limited, substoichiometric amount of [^18^F]fluoride in radiochemistry, a linear correlation between RCC and the extent of protodeboronation is not expected.

### Amount of K_2_CO_3_ as base

Generally, the use of a base such as K_2_CO_3_ has been reported to improve the elution efficiency of [^18^F]fluoride from the anion-exchange cartridge (Tredwell et al. 2014; Zlatopolskiy et al. 2015; Zhou et al. 2022). Moreover, Zhou et al. reported that the application of base plays a critical role in the alcohol-enhanced CMRF in determining the optimum amounts of precursor to be used in the reaction (Zhou et al. 2022). In 1963, a study on the kinetics and the mechanism of the base-catalyzed protodeboronation of areneboronic acids was first reported by Kuivila et al. (1963). Later, several mechanistic investigations were published highlighting the role of the base in catalyzing the formation of HSP of ArB(OH)_2_ in Pd-catalyzed cross-coupling reactions (Cox et al. 2016, 2017; Liu et al. 2015; Lozada et al. 2014). At higher pH, boronic acids are usually present in the boronate form. In 2017, Cox et al. re-investigated the originally described stepwise mechanism (I_A_ in Supporting Information S5) of *ipso* protonation of boronate proposed by Kuivila et al. in 1963 with modern and advanced instrumentation (NMR, stopped-flow IR, and rapid quenched flow) (Cox et al. 2017; Kuivila et al. 1963). It was observed that a concerted mechanism (I_B_ in Supporting Information S5) involving the unimolecular heterolysis of boronate is more favored over the *ipso* protonation of boronate (I_A_ in Supporting Information S5) (Cox et al. 2017). This detailed mechanistic study in organic chemistry provided insights into the involvement of the base in the HSP formation.

**Fig. 6:**
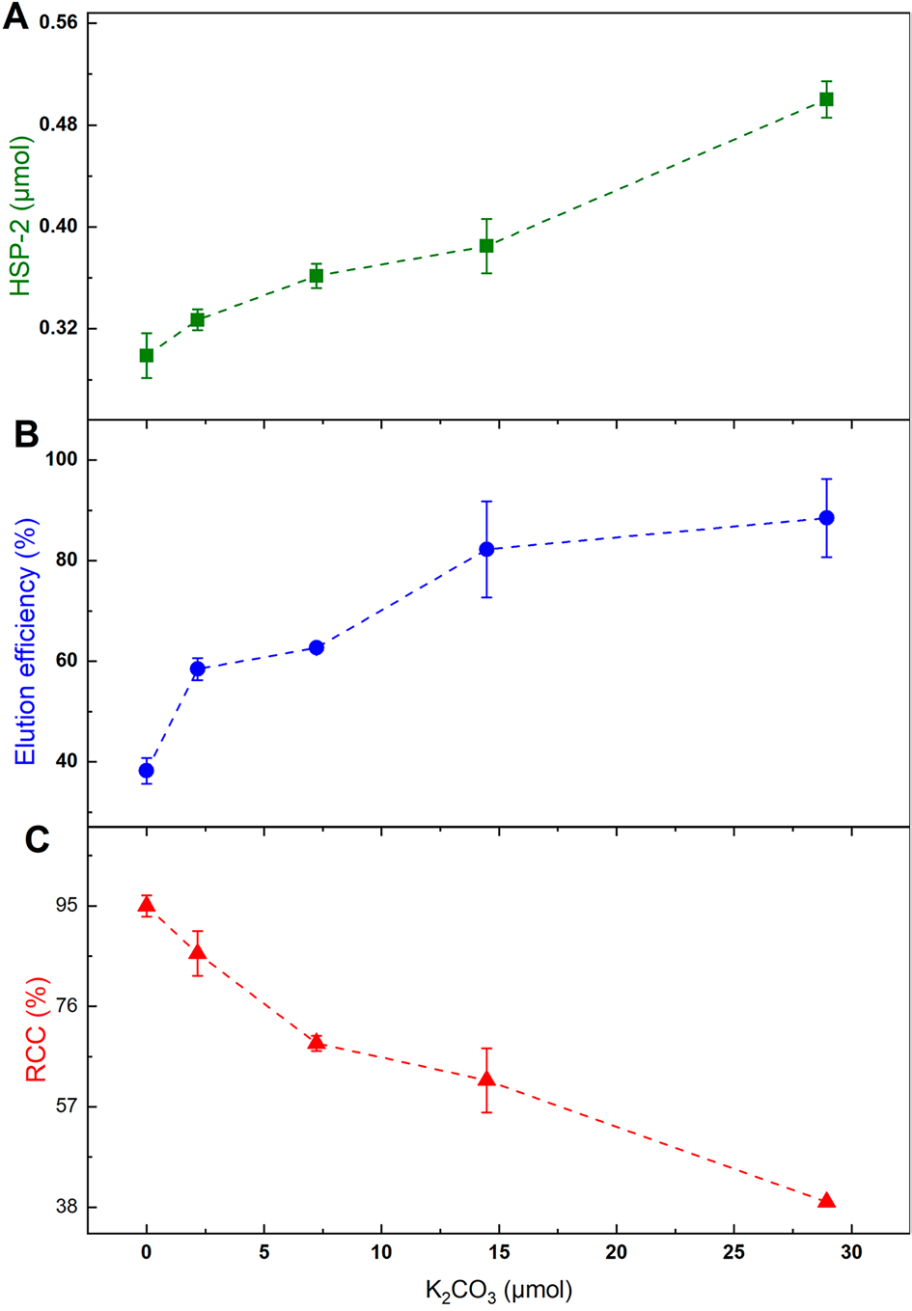
Effect of amount of K_2_CO_3_ (0, 2.2, 7.2, 14.5 and 28.9 µmol) on the **HSP-2** formation (**A**), the release of [^18^F]fluoride trapped on QMA (**B**) and the RCC (**C**) in CMRF of **[**^**18**^**F]2**

Therefore, in the present study, a systematic investigation of the influence of K_2_CO_3_ in CMRF in terms of elution efficiency (also termed as release efficiency) (Herth et al. 2021) of [^18^F]fluoride from QMA, RCC, and **HSP-2** formation was performed using different amounts of K_2_CO_3_ (0, 2.2, 7.2, 14.5 and 28.9 µmol) in combination with TBAHCO_3_ (7.5 mmol) for elution of [^18^F]fluoride from the QMA cartridge. As shown in Fig. 6, the amount of base was positively correlated to both **HSP-2** formation (Graph A) and the release efficiency of [^18^F]fluoride from the QMA cartridge (Graph B), while it inversely affected the RCC of **[**^**18**^**F]2** (Graph C). The exclusion of K_2_CO_3_ resulted in a significant decrease in the release efficiency of [^18^F]fluoride (92% with 28.9 µmol *vs.* 41% without K_2_CO_3_) (Graph B, Fig. 6). Nevertheless, the release efficiency of [^18^F]fluoride from QMA without K_2_CO_3_ can be improved by using a higher amount of TBAHCO_3_ or replacing it with other PTCs such as TEAHCO_3_ or TEAOTf. Additionally, Mossine et al. (2017) developed tailored elution techniques as milder alternatives to K_2_CO_3_ and to minimize or eliminate the need for K222/K_2_CO_3_. Consistent with prior literature, In other words, the exclusion of K_2_CO_3_ as a base could not only decrease the HSP formation but also improve the RCC in the CMRF.

### Acidic protons of precursor

In certain cases, precursors contain acidic protons such as NH and OH groups, which may also be a possible source of protons for the HSP formation in CMRF. To investigate their role, the two NH groups of **7** were deuterated to ND groups by repetitive treatment with MeOD-*d*_*4*_ to get **7**-*d*_*2*_ (Fig. 1 and Supporting Information S1). The CMRF performed with the deuterated **7**-*d*_*2*_ resulted in the formation of a mixture of **DSP-1** and **HSP-1**, demonstrating that the acidic groups, such as NH groups, can actively participate in the HSP formation.

### Reaction temperature

Temperature is a reaction parameter frequently optimized in the CMRF, playing an important role in the reaction kinetics, on RCC, and it is expected that reactions carried out at high temperatures are more prone to give side- or decomposition products that can complicate the purification process.

**Fig. 7:**
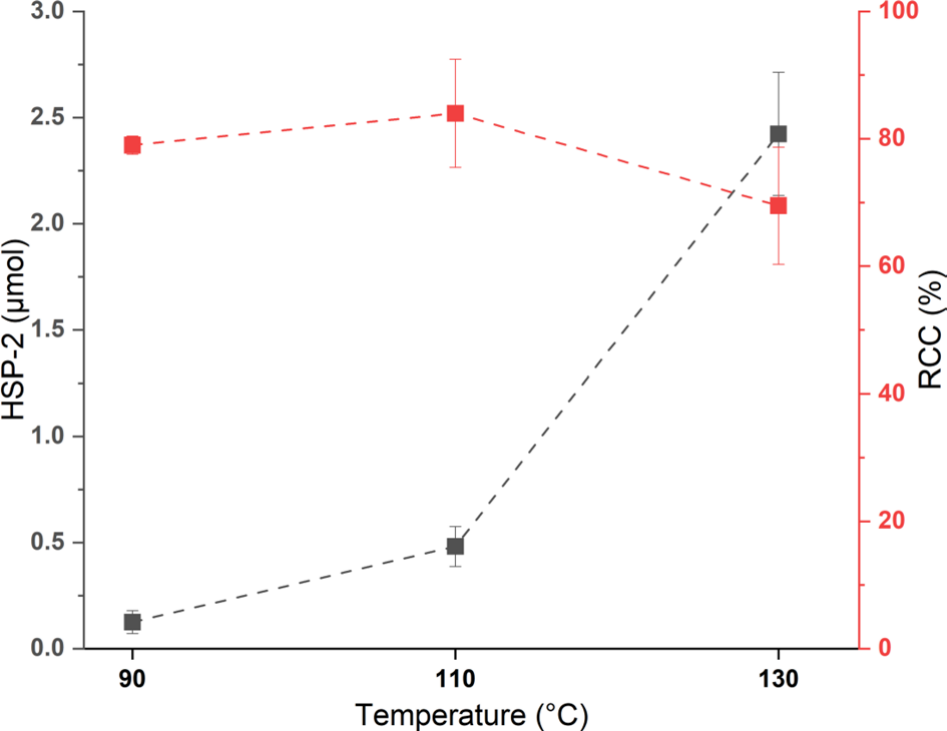
Effect of reaction temperature (90, 110 and 130 °C) on **HSP-2** formation and RCC in CMRF of **[**^**18**^**F]2**

Here, we investigated the effect of temperature on the RCC and HSP formation for CMRF of **[**^**18**^**F]2** using the Bpin precursor **4** at 90, 110 and 130 °C. The RCC and **HSP-2** formation both increased when the temperature was increased from 90 to 110 °C (Fig. 7). However, when the temperature was further increased to 130 °C, RCC decreased with a pronounced increase in the **HSP-2** formation (Fig. 7). This underlines that the reaction temperature in CMRF should be carefully controlled for optimum RCC with minimum HSP formation.

### Amount of copper-complex

Another factor strongly influencing the RCC in CMRFs is the molar amount of the copper-complex (e.g. [Cu(OTf)_2_(Py)_4_]). The use of high amounts of copper-complex might limit the transfer into radiopharmaceutical production of the radiotracer due to purification and toxicity concerns.

**Fig. 8:**
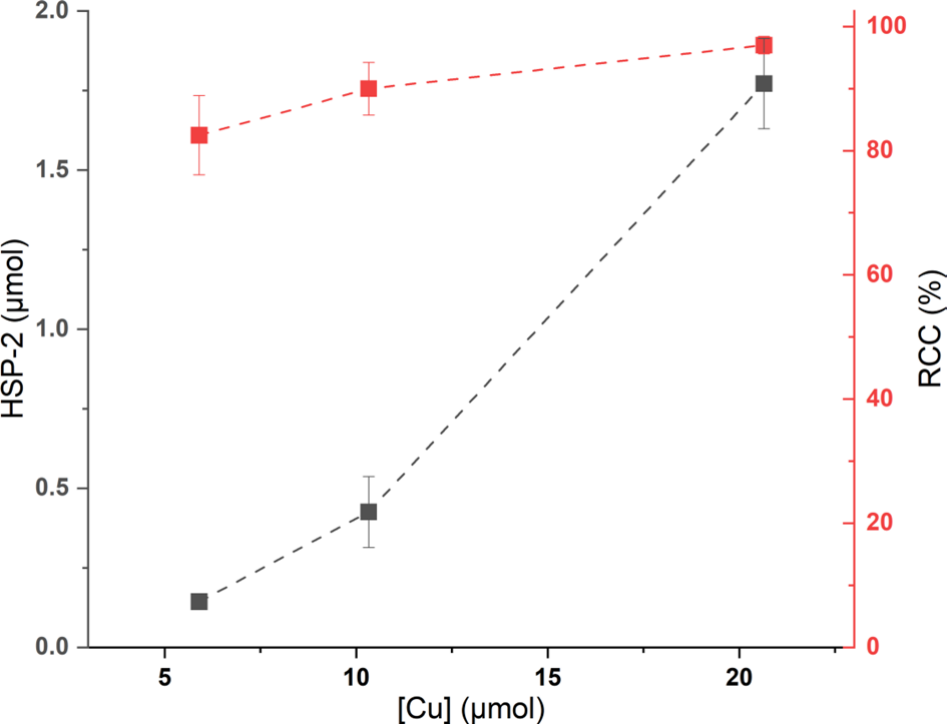
Effect of amount of [Cu(OTf)_2_(Py)_4_] (5.9, 10.3, and 20.7 µmol) on **HSP-2** formation and RCC in CMRF of **[**^**18**^**F]2**

In this study, the impact of the molar amount of [Cu(OTf)_2_(Py)_4_] on HSP formation as well as RCC was investigated. The CMRF of **[**^**18**^**F]2** with different amounts of [Cu(OTf)_2_(Py)_4_] (5.9, 10.3, and 20.7 µmol) was performed and the results showed that the **HSP-2** formation increased significantly with the increase in the amount of [Cu(OTf)_2_(Py)_4_] (Fig. 8). In contrast, the RCC was not significantly affected by the amount of [Cu(OTf)_2_(Py)_4_] used (Fig. 8). Notably, in our study only [Cu(OTf)_2_(Py)_4_] was investigated for its effect on HSP formation. Given the critical role of the copper-complex in radiofluorination and potentially in HSP formation as well, further exploration of alternative copper-complexes is warranted. In this context, Sun et al. recently reported a new protocol utilizing the novel copper-mediator Cu(ONf)_2_, which has shown high efficiency in suppressing protodeboronation (Sun et al. 2024).

## Discussion

In the present study, a systematic investigation of the sources and factors impacting the hydrogenated side product (HSP) formation in CMRF was carried out using two representative radiofluorinated molecules **[**^**18**^**F]1** and **[**^**18**^**F]2**, aiming to support the development of radiopharmaceuticals that are difficult to separate from the corresponding HSP. As the first important finding, it was observed that the aqueous quenching of the reaction mixture does not affect the HSP formation, indicating that the hydrogenation takes place solely during the radiofluorination reaction. However, it cannot be excluded that in certain cases, HSP can occur during the deprotection of precursors. Although the addition of alcohol (*n*-BuOH) as co-solvent enhanced the RCC in our experiments, it also led to a significant increase in the HSP formation, which was not caused by the protons of the alcohol as demonstrated by experiments with fully deuterated *n-*butanol (*n*-BuOD-*d*_*10*_), but rather by the increased reaction kinetics. This observation is in discordance with other studies on cross-coupling reactions involving alcohols and can be explained by the non-stoichiometric reaction conditions and short reaction time of the CMRF. Furthermore, the results showed that excluding K_2_CO_3_ as the base is beneficial for maintaining low HSP formation and improving RCC. Moreover, the stability of the precursor plays an important role and must be inspected before performing the CMRFs, as certain precursor compounds might be prone to degradation to HSP during storage. In our study, -BEpin precursors were evaluated as alternative substrates for CMRF, demonstrating superior chemical stability compared to -Bpin, -B(OH)_2_, and -SnBu_3_ counterparts. Among the compounds tested, the biphenyl BEpin precursor showed the most favorable profile, yielding high RCC with minimal HSP formation, whereas the -B(OH)_2_ resulted in excessively high amounts of HSP. The -Bpin precursors are known to readily hydrolyze under reaction conditions to their corresponding -B(OH)_2_ analogs, which can significantly increase HSP formation. In contrast, -BEpin precursors exhibit enhanced hydrolytic stability, resulting in lower levels of protodeboronation and thus, reduced HSP formation. Moreover, experiments performed with the model compound **[**^**18**^**F]2** showed that, as expected, other reaction parameters influenced not only the RCC but also the formation of the HSP. This trend was observed with factors such as the use of alcohol as a co-solvent, reaction time, reaction temperature, and amount of precursor and [Cu(OTf)_2_(Py)_4_] (Supporting Information S6). Therefore, it is essential to carefully evaluate the balance between RCC and HSP formation. Specifically, reaction conditions like temperature or amount of precursor or amount of [Cu]-reagent that provided only a slight or modest increase in RCC while causing a significant rise in HSP formation should be avoided for CMRF. Conversely, certain reaction conditions demonstrated a detrimental effect on both RCC and HSP formation (decreasing RCC with increasing HSP formation). Examples include the use of B(OH)_2_-based precursors, high amounts of K_2_CO_3_ as base, high temperatures, and excessive amounts of precursor and [Cu(OTf)_2_(Py)_4_] (Supporting Information S6). In practical reaction systems, the formation of HSP can result from the synergistic effects of multiple reaction parameters or sources. These may include temperature, solvent effects, catalyst choice, reagent purity, precursor stability, or reaction time, which collectively or independently contribute to side product pathways. From the results obtained in the above section, four different reaction conditions, (A, B, C and D) (Table 1) were selected and compared, highlighting that fine-tuning these parameters can markedly reduce undesirable HSP formation while maintaining or improving RCC. Specifically, protocol D using mild reaction conditions and reduced amount of reagents yielded only ~ 0.02 µmol of **HSP-2** (0.4% yield) with an acceptable RCC of 58% as compared to e.g. protocol A which yielded 4.5 µmol of **HSP-2** (60% yield) with a similar RCC of 50%, demonstrating the value of integrated optimization of various reaction parameters. However, in other cases of reaction optimization for radiopharmaceutical production, individual decisions have to be taken to achieve a balanced HSP to RCC ratio depending on demand.

**Table 1:** Comparison of reaction protocols A, B, C and D and the impact on the RCC and **HSP-2** formation through the CMRF of **[**^**18**^**F]2**

Reaction protocol → Reaction parameter ↓	A	B	C	D
Precursor (µmol)	7.5	7.5	7.5	5
^18^F source	[^18^F]TBAF	[^18^F]TBAF	[^18^F]TBAF	[^18^F]TEAF^a^
Cu(OTf)_2_(Py)_4_ (µmol)	10.3	10.3	10.3	6
Solvent (900 µL)^b^	DMI	DMI:*n*-BuOH 2:1	DMI	DMI
K_2_CO_3_ (µmol)	2	0	0	0
Temperature (°C)	110	110	110	90
Time (min)	10	10	10	2
RCC (%)	50	97	90	58
**HSP-2** (µmol)	4.5	1.8	0.5	0.02
**HSP-2** (%)^c^	60	24	7	0.4

^a^Although the influence of TBAHCO_3_ and TEAHCO_3_ on the **HSP-2** formation was insignificant, we have selected TEAHCO_3_ because it is inexpensive as compared to TBAHCO_3_

^b^Total reaction volume is 900 µL

^c^Percentage yield of **HSP-2** compared to precursor used

The two compounds evaluated in the present study are belonging to different structural classes, and this might influence the mechanistic study presented herein. However, besides the acidic protons of compound **[**^**18**^**F]1**, which can act as a source of hydrogen, we do not observe any other factors that might influence the mechanistic study. While the heteroatoms of compound [^**18**^**F]1** might coordinate with [Cu], thereby influencing the reaction rate, under standard reaction conditions, a similar radiolabeling profile can be observed for both classes of compound. Therefore, we can assume that in our case the heteroatoms are not influencing the reaction rate. Furthermore, the electronic density at the investigated position is similar for both compounds and should not affect the mechanistic evaluation presented in this manuscript either. Thus, we conclude that the two compounds evaluated herein demonstrate similar radiochemical reactivity and are suitable examples for the present study. Although the present study was carried out for a small number of molecules, our results can be used as a starting point for optimizing the reaction conditions and selecting a suitable precursor. Nevertheless, the evaluation of alternative aprotic solvents, new types of precursors, copper complexes, and fluorinating agents is warranted. The CMRF is rapidly expanding in automated radiopharmaceutical production applications, which emphasizes the need for further developments and improvements to successfully transfer manual radiosynthesis into radiosynthesizer-based automated radiopharmaceutical production, including the solution of challenges such as hydrogenation side-reaction.

## Conclusion

Overall, this study reports on the effect of various parameters on the hydrogenation side reaction in relation to RCC. It provides insights into reaction conditions that should be avoided or carefully controlled for achieving an optimal RCC while suppressing HSP formation in CMRF. Thus, to minimize HSP formation, the reaction conditions should be kept as mild as possible by using a low temperature, a short reaction time, a small amount of precursor, base and copper. Furthermore, although the use of alcohol increases the reaction kinetics, it can lead to excessive HSP formation. Among the precursors, -BEpins gives the lowest amount of HSP, whereas -B(OH)_2_ gives the highest. Implementing the optimal reaction conditions in an automated radiosynthesis procedure should pose no challenge, except for the short reaction time (e.g. 2 min), which may be difficult. Further investigations are underway, including the use of different precursor compounds, Cu-mediators, additives, and substrates, as well as mechanistic studies.

## Methods

### Synthesis of precursors

All chemicals and reagents were commercially purchased and used without further purification unless otherwise mentioned. Moisture-sensitive reactions were carried out under dry argon. The solvents used were purified and dried according to standard procedures. Distilled solvents of technical grade were used for purification procedures. Unless otherwise stated, the yields correspond to the purified compounds. ^1^H, ^13^C, ^11^B and ^119^Sn NMR spectra were measured on VARIAN “MERCURY plus” (300 MHz for ^1^H-NMR, 75 MHz for ^13^C-NMR, 282 MHz for ^19^F-NMR) and BRUKER DRX-400 (400 MHz for ^1^H-NMR, 100 MHz for ^13^C-NMR, 377 MHz for ^19^F-NMR). The chemical shifts (*δ*) of signals are reported in ppm units. All spectra were recorded at r.t. followed by calibration on the solvent signal [CDCl_3_: *δ* (^1^H-NMR) = 7.26 ppm and *δ* (^13^C-NMR) = 77.16 ppm] and on TMS as an external standard. Multiplicities of NMR (J) signals are indicated as follows: s (singlet), d (doublet), t(triplet), q (quartet), m (multiplet), dd (doublet of doublets), dt (doublet of triplets), td (triplet of doublets), ddd (doublet of doublets of doublets).

LC–MS was performed on a Dionex Ultimate 3000 system, incorporating an LPG-3400SD pump, an autosampler WPS-3000 TSL, a column compartment TCC-3000SD, a diode array detector DAD3000 (monitoring from 254 to 720 nm) and a low-resolution mass spectrometer MSQ 3000 (Thermo Fisher Scientific Inc., Waltham, USA).

Analytical chromatographic separations were performed on a JASCO LC-2011 system, incorporating a PU-2080Plus pump, AS-2055Plus auto-injector (100 μL sample loop), and a UV-2070Plus detector (Jasco Deutschland GmbH, Pfungstadt, Germany) coupled with an optical activity HPLC detector. Data analysis was performed with the Galaxie chromatography software (Agilent Technologies).

Analytical thin-layer chromatography was performed on silica gel-coated plates (Macherey–Nagel, ALUGRAM SIL G/UV254). The spots were identified by using a UV lamp or by a suitable TLC staining system (iodine vapor, 0.1% ninhydrin in ethanol/water 1/10 (*v/v*), 10% phosphomolybdic acid in ethanol, KMNO_4_ solution in water, Hanessian's Stain).

The flash column chromatography was performed on silica gel ZEOsorb 60/40–63 µm from Apollo Scientific Ltd. and silica gel 40–63 µm from VWR Chemicals. The chemical purity of final compounds (≥ 95%) was controlled by LC–MS using a Reprosil-Pur Basic HD column (150 × 3 mm, 3 µm, Dr. Maisch GmbH, Germany). A mixture of MeCN and 20 mM NH_4_OAc_aq_ was used as eluent in a linear gradient system with a flow of 0.7 mL/min. The ammonium acetate concentration stated as 20 mM NH_4_OAc_aq_ corresponds to the concentration in the aqueous component of an eluent mixture.

Chemical names, chemical formulas, and exact mass of compounds were generated by ChemDraw Professional 17.0.

#### General procedure I

A flask with a magnetic stirrer was charged with **5** (1 eq) in dry THF under argon at r.t. Afterwards, Cs_2_CO_3_ (4 eq) was added and stirred for 1 h at r.t. Successively, respective aryl bromide (1.2 eq) was added and the reaction mixture was stirred overnight (15 h) at r.t. under argon. The reaction mixture was rotary evaporated to remove THF and then extracted with two portions of EtOAc and water. The combined extracts were washed with brine, dried over MgSO_4_, filtered and concentrated under reduced pressure. The residue was purified by silica gel chromatography (increasing gradient of PE/EtOAc). The appropriate fractions were combined, and concentrated under reduced pressure and the residue was dried to constant weight under a high vacuum to provide the respective product.

#### 3, 7,7-dimethyl-5-(1H-pyrrole-2-carbonyl)-1-(4-(4,4,5,5-tetramethyl-1,3,2-dioxaborolan-2-yl)benzyl)-N-(m-tolyl)-4,5,6,7-tetrahydro-1H-pyrazolo[4,3-c]pyridine-3-carboxamid, C_34_H_40_BN_5_O_4_

General procedure I was followed starting with 350 mg of **5** (0.93 mmol, 1 eq): Yield 48%. ^1^H NMR (400 MHz, CDCl_3_) δ ppm 9.47 (s, NH), 8.59 (s, NH), 7.77 (d, *J* = 8.0 Hz, 2H), 7.50 (s, 1H), 7.43 (dd, *J* = 8.0, 2.2 Hz, 1H), 7.23 (t, *J* = 7.8 Hz, 1H), 7.03 (d, *J* = 7.8 Hz, 2H), 6.96–6.90 (m, 2H), 6.88 (s, 1H), 6.30 (q, *J* = 3.0 Hz, 1H), 5.48 (s, 2H), 5.26 (s, 2H), 3.74 (s, 2H), 2.36 (s, 3H), 1.33 (s, 12H), 1.24 (s, 6H). ^13^C NMR (101 MHz, CDCl_3_) δ ppm 162.33, 160.50, 140.97, 139.93, 139.26, 137.92, 135.60, 129.16, 125.85, 125.24, 124.91, 121.35, 120.72, 117.23, 115.70, 113.69, 110.60, 84.25, 75.35, 55.53, 34.29, 30.01, 25.39, 25.17, 21.82. ^11^B NMR (128 MHz, CDCl_3_) δ ppm 31.81. HRMS (ESI +): *m*/*z* (%) = 594.3236, calculated 594.3173 for C_34_H_41_BN_5_O_4_ [M + H]^+^.

#### 6, 1-(4-iodobenzyl)-7,7-dimethyl-5-(1H-pyrrole-2-carbonyl)-N-(m-tolyl)-4,5,6,7-tetrahydro-1H-pyrazolo[4,3-c]pyridine-3-carboxamide, C_2_8H_28_IN_5_O_2_

General procedure I was followed starting with 200 mg of **5** (0.53 mmol, 1 eq): Yield 78%. ^1^H NMR (400 MHz, CDCl_3_) δ ppm 9.44 (s, NH), 8.55 (s, NH), 7.67 (d, *J* = 8.4 Hz, 2H), 7.50 (s, 1H), 7.42 (d, *J* = 8.4 Hz, 1H), 7.23 (t, *J* = 7.8 Hz, 1H), 6.94 (ddq, *J* = 5.2, 2.5, 1.1 Hz, 2H), 6.88 (s, 1H), 6.82–6.74 (m, 2H), 6.31 (dt, *J* = 3.8, 2.7 Hz, 1H), 5.40 (s, 2H), 5.25 (s, 2H), 3.75 (s, 2H), 2.37 (s, 3H), 1.26 (s, 6H). ^13^C NMR (101 MHz, CDCl_3_) δ ppm 162.00, 160.01, 146.95, 140.87, 138.97, 137.96, 137.52, 136.33, 128.86, 128.16, 125.00, 124.52, 121.11, 120.38, 116.90, 115.53, 113.38, 110.29, 93.36, 54.55, 44.19, 33.96, 25.16, 21.50. HRMS (ESI +): *m*/*z* (%) = 594.1358, calculated 594.1288 for C_28_H_29_IN_5_O_2_ [M + H]^+^.

#### 7, 7,7-dimethyl-5-(1H-pyrrole-2-carbonyl)-N-(m-tolyl)-1-(4-(tributylstannyl)benzyl)-4,5,6,7-tetrahydro-1H-pyrazolo[4,3-c]pyridine-3-carboxamide, C_40_H_55_N_5_O_2_Sn

The compound **6** (40 mg, 0.067 mmol) was dissolved in anhydrous propan-2-ol (2 mL) under argon flow. Afterwards, *tris*(dibenzylidenacetone)palladium(0) (1 mg, 0.002 mmol), hexabutylditin (40 µL, 0.09 mmol), and DIPEA (97 µL, 0,268 mmol) were added. The reaction mixture was then stirred at r.t. for 15 h, the mixture was filtered on Celite 545 and washed with EtOAc (3 × 50 mL). After evaporation of the filtrate under reduced pressure, the crude residue was purified by column chromatography (SiO_2_, cyclohexane/EtOAc, 5/1, *v/v* and then cyclohexane/EtOAc, 5/6, *v/v*) to provide derivative **7** (38 mg, 0.05 mmol, 75%) as a light yellow solid to be stored at 4 °C under argon atmosphere and protected from light. ^1^H NMR (400 MHz, CDCl_3_) δ ppm 9.50 (s, NH), 8.63 (s, NH), 7.54 (s, 1H), 7.45 (d, *J* = 7.6 Hz, 2H), 7.26 (t, *J* = 8.3 Hz, 1H), 7.01 (d, *J* = 7.3 Hz, 2H), 6.98–6.93 (m, 2H), 6.91 (s, 1H), 6.33 (q, *J* = 3.0 Hz, 1H), 5.48 (s, 2H), 5.29 (s, 2H), 3.77 (s, 2H), 2.39 (s, 3H), 1.54 (dq, *J* = 12.9, 7.8 Hz, 6H), 1.33 (q, *J* = 7.4 Hz, 14H), 1.28 (s, 6H), 1.16–0.95 (m, 6H), 0.89 (t, *J* = 7.3 Hz, 9H). ^13^C NMR (75 MHz, CDCl_3_) δ 162.61, 160.79, 147.41, 142.43, 141.06, 139.52, 138.21, 137.44, 136.76, 129.42, 126.31, 125.48, 125.16, 121.65, 120.96, 117.47, 115.93, 113.99, 110.85, 55.76, 34.57, 30.27, 29.74, 29.61, 27.88, 25.59, 22.09, 14.22, 10.16. ^119^Sn NMR (149 MHz, CDCl_3_) δ ppm -40.87. HRMS (ESI +): *m*/*z* (%) = 758.3436, calculated 758.3378 for C_40_H_56_N_5_O_2_Sn [M + H]^+^.

#### 8, [1,1'-biphenyl]-4-yltributylstannane, C_24_H_36_Sn

A solution of *n*-BuLi (2.5 M in hexane, 0.72 mmol, 1 eq) was slowly added to a solution of 4-Iodobiphenyl (200 mg, 0.72 mmol, 1 eq) in THF at − 78 °C and stirred for 30 min. Afterwards, Bu_3_SnCl (234.36 mg, 0.72 mmol, 1 eq) was added slowly at − 78 °C and stirred for 1 h. Then the reaction was brought to r.t. slowly. Then the solvent was removed under reduced pressure, and the resulting residue was dissolved in 20 mL of Et_2_O. The residue was filtered through a plug of neutral alumina and the filtrate concentrated under reduced pressure to get a viscous colorless oil **8** (220 mg, 0.50 mmol, 69%). ^1^H NMR (400 MHz, CDCl_3_) δ ppm 7.67–7.40 (m, 8H), 7.40–7.31 (m, 1H), 1.67–1.49 (m, 6H), 1.42–1.32 (m, 6H), 1.20–0.99 (m, 6H), 0.91 (t, *J* = 7.3 Hz, 9H). ^13^C NMR (101 MHz, CDCl_3_) δ 141.47, 141.01, 140.89, 137.19, 137.04, 136.89, 128.89, 128.86, 127.31, 127.25, 126.76, 29.27, 27.55, 13.83, 9.78. ^119^Sn NMR (149 MHz, CDCl_3_) δ ppm 106.47, − 41.48. It was noted that the **8** was highly unstable on silica, in water, protic solvents, and degrades to biphenyl (**HSP-2**).

#### 9, 2-([1,1'-biphenyl]-4-yl)-4,4,5,5-tetraethyl-1,3,2-dioxaborolane, C_22_H_29_BO_2_

A flask with a magnetic stirrer was charged with 4-biphenyl boronic acid **11** (100 mg, 0.50 mmol, 1.0 eq), and 3,4-diethylhexane-3,4-diol (174.28 mg, 0.50 mmol, 1.0 eq) and evacuated to remove air and backfilled with Ar. Anhydrous CH_2_Cl_2_ was added to the flask with a syringe and the mixture was stirred for 4 h at r.t. After the completion of the reaction, the reaction was quenched with 0.5 mL of water. The resulting reaction mixture was extracted with 3 × 50 mL CH_2_Cl_2_, the combined organic phases were dried over anhydrous MgSO_4_ and the solvent was removed under reduced pressure to get a crude product. The crude product was purified by flash chromatography on silica gel with isocratic hexane/EtOAc, 19/1, *v/v* to get colorless oil **9** (148.1 mg, 0.440 mmol, Yield 87%). ^1^H NMR (400 MHz, CDCl_3_) δ ppm 7.91 (d, *J* = 8.4 Hz, 2H), 7.66–7.56 (m, 4H), 7.49–7.40 (m, 2H), 7.39–7.31 (m, 1H), 1.79 (dtq, *J* = 21.6, 14.6, 7.4 Hz, 8H), 0.99 (t, *J* = 7.5 Hz, 12H). ^13^C NMR (101 MHz, CDCl_3_) δ 143.91, 141.31, 135.45, 128.91, 127.65, 127.40, 126.60, 88.95, 26.64, 9.02. ^11^B NMR (128 MHz, CDCl_3_) δ ppm 32.05.

### Radiofluorination

#### General

The precursors **4** and **11** were commercially procured from Sigma-Aldrich Chemie GmbH, Part of Merck, Taufkirchen, Germany. The precursors **3**, **7**, **8**, and **9** were synthesized as mentioned in Supporting Information S1. The reference compound for **HSP-1** was synthesized as mentioned in the literature (Kaur et al. 2025) and for **HSP-2** was purchased from Sigma-Aldrich Chemie GmbH, Part of Merck, Taufkirchen, Germany.

The anhydrous labeling solvents 1,3-dimethyl-2-imidazolidinone (DMI) and *n*-butanol (*n-*BuOH), were purchased from Sigma-Aldrich Chemie GmbH, Part of Merck, Taufkirchen, Germany. The *n-*tetrabutylammonium hydrogencarbonate (TBAHCO_3_) was used as a 0.075 M solution provided by ABX advanced biochemical compounds GmbH, Radeberg, Germany. The *n-*tetraethylammonium hydrogencarbonate (TEAHCO_3_) was purchased from Sigma-Aldrich Chemie GmbH, Part of Merck, Taufkirchen, Germany. The deuterated solvents *n-BuOD-d*_*10*_ (99 atom% D) and D_2_O (99.8 atom% D) were purchased from abcr GmbH, Germany. The *n-*tetrabutylammonium deuteriumcarbonate (TBADCO_3_) was prepared by isotope exchange by repetitive treatment of TBAHCO_3_ with an excess of D_2_O for deuterated experiments.

Radio thin layer chromatography (radio-TLC) was performed on silica gel (Polygram® SIL G/UV254 from Machery-Nagel, Germany) pre-coated plates with a mixture of EtOAc/*n*-hexane 3/1 *(v/v)* as eluent. The plates were exposed to storage phosphor screens (BAS-IP MS 2025, FUJIFILM Co., Tokyo, Japan) and recorded using the Amersham Typhoon RGB Biomolecular Imager (GE Healthcare Life Sciences). Images were quantified with the ImageQuant TL8.1 software (GE Healthcare Life Sciences).

Analytical radio-HPLC was performed on a JASCO LC-2000 system, incorporating a PU-2080Plus pump, AS-2055Plus auto-injector (100 μL sample loop), and a UV-2070Plus detector (Jasco Deutschland GmbH, Pfungstadt, Germany) coupled with a gamma radioactivity HPLC detector (Gabi Star, Elysia-raytest GmbH, Straubenhardt, Germany). Data analysis was performed with the Galaxy chromatography software (Agilent Technologies).

High-resolution mass spectra (HRMS) were recorded in flow injection mode on an ESI-TOF micrOTOF (Bruker Daltonics; Bruker Corporation, Billerica, MA, USA), with an Agilent 1100 series HPLC pump and autosampler, operated by OtofControl 3.4 und HyStar 3.2-LC/MS. Data was acquired in fullscan positive ion mode between *m/z* 100–2000, with a nebulizer pressure at 2.2 bar, dry gas flow at 6 L/min (both nitrogen) and dry gas temperature at 220 °C. Voltages were adjusted for capillary to 4.5 kV, end plate offset − 0.5 kV, cap exit 150 V, and hexapole RF to 350 Vpp; extraction trigger time and pulse width were set to 89 µs and 5 µs, respectively.

For the quantification of **HSP-1**/**HSP-2**, a Nucleodur PFP from Macherey–Nagel, 250 × 4.6 mm, 5 µm and for HPLC purification, a ReproSil Fluosil 100 PFP column (250 × 10 mm; 5 µm; Dr. Maisch HPLC GmbH; Germany) with MeCN/20 mM NH_4_OAc_aq_. (pH 6.8) as the eluent mixture under the conditions mentioned below in Table 2 was used (Details in Supporting Information S3).

**Table 2:** HPLC conditions used for the analytical investigations of **HSP-1** and **HSP-2**

Compound	Stationary phase	Mobile phase	*t*_R_ (min)
*Analytical HPLC conditions*			
**HSP-1**	Nucleodur PFP, 250 × 4.6 mm, 5 µm	52% MeCN/20 mM NH_4_OAc_aq_, 1 mL/min, 268 nm	17.2
**HSP-2**	Nucleodur PFP, 250 × 4.6 mm, 5 µm	50% MeCN/20 mM NH_4_OAc_aq_, 1 mL/min, 254 nm	21.0
*Semi-preparative HPLC conditions*			
**HSP-1**	ReproSil Fluosil 100 PFP, 250 × 10 mm; 5 µm	52% MeCN/H_2_O, 4 mL/min, 268 nm	~ 27
**HSP-2**	ReproSil Fluosil 100 PFP, 250 × 10 mm; 5 µm	54% MeCN/H_2_O, 4 mL/min, 254 nm	~ 21

#### [^18^F]Fluoride, [^18^F]TEAF, and [^18^F]TBAF production

No-carrier-added [^18^F]fluoride was produced via the ^18^O(*p,n*)^18^F nuclear reaction by irradiation of an [^18^O]H_2_O liquid target (Hyox 18 enriched water, Rotem Industries Ltd, Israel) on a Cyclone 18/9 (iba RadioPharma Solutions, Belgium) with fixed energy proton beam using Nirta [^18^F]fluoride XL target.

Preconditioning of Sep-Pak® Accell QMA light cartridge (Waters GmbH, Eschborn, Germany) was done using 10 mL of 0.5 M NaHCO_3_ followed by 10 mL water. Preconditioning of Sep-Pak® C18 plus cartridges (Waters GmbH, Eschborn, Germany) was done using 5 mL of EtOH and 20 mL of water. For experiments with deuterated solvents, these cartridges were additionally washed with 200 µL of D_2_O.

For the production of [^18^F]TBAF (*n-*tetrabutylammonium fluoride), no carrier-added (n.c.a) [^18^F]fluoride was trapped on the pre-conditioned cartridge Sep-Pak® Accell QMA light cartridge (Waters GmbH, Eschborn, Germany). The loaded [^18^F]fluoride was eluted with a mixture of 800 µL of MeCN, 100 µL of 0.075 M solution of TBAHCO_3_ and 100 µl of water. The eluted aqueous [^18^F]fluoride was azeotropically dried under vacuum and nitrogen flow within 7–10 min using a single mode microwave (75 W, at 50–60 °C, power cycling mode; Discover PETWave from CEM corporation, USA). Two aliquots of MeCN (2 × 1.0 mL) were added during the drying procedure and the final ^18^F-reagent was obtained as a white solid.

For the production of [^18^F]TEAF (*n-*tetraethylammonium fluoride), the same procedure as for the production of [^18^F]TBAF was used with slight modifications. Briefly, n.c.a [^18^F]fluoride trapped on the pre-conditioned cartridge Sep-Pak® Accell QMA light cartridge was eluted with a mixture of 800 µL of MeCN, 100 µL of 0.075 M solution of TEAHCO_3_ and 100 µl of water. The eluted aqueous [^18^F]fluoride was azeotropically dried as mentioned above.

For the production of [^18^F]TBAF from TBAOTf, n.c.a [^18^F]fluoride in 1.0 mL of water was trapped on a preconditioned cartridge Sep-Pak® Accell QMA light cartridge. After loading, the cartridge was eluted with a mixture of 200 µL of MeCN, 100 µL of 0.075 M solution of TBAOTf, 200 µL of water and 200 µL of MeOH. The eluted aqueous [^18^F]fluoride was azeotropically dried as mentioned above.

#### General procedure II: Manual synthesis of [^18^F]1 and [^18^F]2

The azeotropically dried [^18^F]TBAF (prepared from TBAHCO_3_) was dissolved in the solvent (300 µL of DMI), followed by addition of [Cu(OTf)_2_(Py)_4_] (15 µmol for **[**^**18**^**F]1** and 10.3 µmol for **[**^**18**^**F]2**) in 300 µL of DMI, pre-stirred for 2 min at r.t., then the respective precursor (3.4 µmol of **3** or 7.1 µmol of **4**) (300 µL of DMI) was added and the resulting reaction mixture was stirred at 110 °C for 10 min. The reaction mixture was cooled to r.t. and the RCC was determined by radio-TLC and periodically by radio-HPLC. For quantitative analysis of HSP, an aliquot of the reaction mixture (30 µL, 1–5 MBq) was diluted to 100 µL with MeCN/ H_2_O 1/1 (*v/v*) and analyzed by an analytical HPLC under respective isocratic conditions (Table 2). The amount of **HSP-1**/**HSP-2** was calculated from a calibration curve obtained under the same HPLC conditions (Supporting Information S3).

The following possible source(s) and reaction parameters were systematically investigated for the formation of **HSP-1**/**HSP-2** through the CMRF of **[**^**18**^**F]1**/**[**^**18**^**F]2**: *(i) aqueous quenching of the reaction mixture; (ii) alcohol as co-solvent; (iii) different phase-transfer catalysts (PTCs); (iv) reaction time; (v) different leaving groups of precursor; (vi) amount of precursor; (vii) amount of base (K*_*2*_*CO*_*3*_*); (viii) acidic protons of precursor; (ix) reaction temperature; and (x) molar amount of copper-complex.*

The general procedure (II) was modified according to the investigating parameter as mentioned below in detail. In certain experiments, deuterated reagents (D_2_O, *n*-BuOD-*d*_*10*_, **7**-*d*_*2*_) were used in the radiofluorinations to determine the source hydrogen responsible for the **HSP** formation. In these deuterated experiments, the reaction mixture was quenched with 3 mL of D_2_O and loaded on the pre-conditioned Sep-Pak® C18 plus cartridge, then 1 mL of air and then the product was eluted with 2.5 mL of MeCN and further diluted with 2.5 mL of D_2_O. This final 5 mL eluent was subjected to HPLC purification of **DSP-1**/**HSP-1** under the above-mentioned conditions in Table 2. The fraction containing the **DSP-1**/**HSP-1** was collected, under reduced vacuum, and investigated with high-resolution mass spectrometry (HRMS) to determine the extent of deuteration.

#### Aqueous quenching of reaction mixture

To investigate the role of aqueous quenching, the general procedure II mentioned above was employed with slight deviations to produce **[**^**18**^**F]1**. For the reaction quenched with D_2_O, [^18^F]TBAF was prepared from TBADCO_3_ in D_2_O (as mentioned above) and the cartridges were additionally conditioned with D_2_O (200 µL). Briefly, to the pre-stirred solution mixture of [^18^F]TBAF (630 MBq) and 15 µmol of [Cu(OTf)_2_(Py)_4_] in 600 µL of DMI, 3.4 µmol of **3** in 300 µL of DMI was added and the resulting reaction mixture was stirred at 110 °C for 10 min. The 30 µL aliquot of the reaction mixture (further diluted with 70 µL MeCN/ H_2_O 1/1 (*v/v*) was taken for analytical investigation. The cooled reaction mixture was divided into two parts of 450 µL each (approximately 250 MBq each), one part was quenched with 3 mL D_2_O and the other one with 3 mL H_2_O. The quenched reaction mixture was loaded on preconditioned Sep-Pak® C18 plus cartridges, the loaded **[**^**18**^**F]1** was eluted with 2.5 mL of MeCN and further diluted with corresponding 2.5 mL of D_2_O or H_2_O. The final 5 mL eluent was injected into the HPLC column under the conditions mentioned in Table 2. The corresponding product (**DSP-1** or **HSP-1**) was collected and concentrated under a reduced vacuum to investigate the level of deuteration by HRMS. This experiment was done in triplicate (n = 3).

#### Alcohol as co-solvent

To investigate the role of alcohol as a solvent, the general procedure II mentioned above was employed with slight deviations to produce **[**^**18**^**F]1**. Three radiofluorination reactions were done simultaneously, differing in the solvent mixture (DMI, or DMI/*n*-BuOH 2/1 (*v/v*), or DMI/*n-BuOD-d*_*10*_ 2/1 (*v/v*)). Briefly, the azeotropically dried [^18^F]fluoride (1400 MBq) was dissolved in 900 µL of DMI, followed by the addition of 45 µmol [Cu(OTf)_2_(Py)_4_] in 900 µL of DMI. The resulting mixture was stirred for 2 min at r.t. and then it was divided into three 600 µL portions (392–400 MBq each) and transferred to separate reaction vials. Subsequently, 3.4 µmol of **3** in 300 µL of DMI or *n*-BuOH or *n-BuOD-d*_*10*_ was added into respective vials and stirred for 10 min at 110 °C. The 30 µL aliquot of the reaction mixture was diluted to 100 µL with MeCN/ H_2_O 1/1 (*v/v*) to investigate the HSP formation. To investigate the deuterium incorporation, the reaction mixtures were then quenched with the 3 mL H_2_O or D_2_O (for reaction with *n-BuOD-d*_*10*_). Each quenched reaction mixture was loaded on preconditioned Sep-Pak® C18 plus cartridges, eluted with 2.5 mL of MeCN and further diluted with corresponding 2.5 mL of D_2_O or H_2_O. The final 5 mL eluent was injected into the HPLC column under the conditions mentioned in Table 2. The corresponding product (**DSP-1** or **HSP-1**) was collected and concentrated under a reduced vacuum to investigate the extent of deuteration with HRMS. This experiment was done in triplicate (n = 3).

#### Different phase-transfer catalysts and reaction time

The influence of different PTCs (TBAHCO_3_ or TBAOTf or TEAHCO_3_) and reaction time (2, 5, 10, 15, 20, and 30 min) was carried out in parallel. Three radiofluorination reactions were carried out using azeotropically dried [^18^F]TBAF (prepared from TBAHCO_3_) or [^18^F]TBAF (prepared from TBAOTf) or [^18^F]TEAF (prepared from TEAHCO_3_). The preparation of azeotropically dried [^18^F]TBAF, [^18^F]TEAF from TBAHCO_3_ or TBAOTf or TEAHCO_3_ respectively is described above. For each of the reaction, the azeotropically dried [^18^F]fluoride (250–350 MBq each) was dissolved in 300 µL of DMI each, followed by addition of 10.3 µmol [Cu(OTf)_2_(Py)_4_] (300 µL of DMI), stirred for 2 min at r.t., then 7.1 µmol of **4** in 300 µL of DMI was added and stirred at 110 °C. The 30 µL aliquots of the reaction mixture were taken at 2, 5, 10, 15, 20 and 30 min to quantitatively investigate the **HSP-2** formation on the analytical HPLC (n = 2).

#### Different leaving groups of precursor

The influence of precursor types (-B(OH)_2_, -BPin, -BEpin, and -SnBu_3_) on HSP formation was evaluated through four concurrent CMRFs of **[**^**18**^**F]2** with precursors **4** or **8** or **9** or **11**. The radiosynthesis of **[**^**18**^**F]2** was conducted following the general procedure II, differing in the type of precursor used, to assess its impact on the RCC and **HSP-2** formation. Approximately 1.3 GBq of azeotropically dried [^18^F]TBAF was resolubilized in 1200 µL of DMI, combined with 41.2 µmol of [Cu(OTf)_2_(Py)_4_] in 1200 µL of DMI, and stirred at r.t. for 2 min. This solution was divided into four 600 µL portions (270–273 MBq each) and transferred to separate reaction vials. Afterwards, 7.1 µmol of compounds **4** or **8** or **9** or **11** in 300 µL of DMI was introduced into the respective vials. The mixtures were stirred at 110 °C for 10 min. After the reaction, aliquots of each reaction were taken and the **HSP-2** formation was investigated analytically in each reaction. This was investigated thrice (n = 3).

The effect of -SnBu_3_ *vs.* -BPin precursors on the HSP formation was also compared through two CMRFs of **[**^**18**^**F]1**, with precursors **7** or **3**, keeping the rest of the reaction conditions the same as mentioned in the general procedure II. Approximately 750 MBq of azeotropically dried [^18^F]TBAF was resolubilized in 600 µL of DMI, combined with 30 µmol of [Cu(OTf)_2_(Py)_4_] in 600 µL of DMI, and stirred at r.t. for 2 min. This solution was divided into two 600 µL portions (349 and 347 MBq) and transferred to separate reaction vials. Afterwards, 3.4 µmol of compounds **3** or **7** in 300 µL of DMI was introduced into the respective vials. The mixtures were stirred at 110 °C for 10 min. After the reaction, the formation of **HSP-1** was quantified similarly as mentioned above.

#### Amount of precursor

The radiosynthesis of **[**^**18**^**F]2** was conducted following the general procedure II, with modifications in the quantity of precursor **4** to assess its impact on the reaction outcome. Four concurrent CMRF reactions were performed using 3.6, 7.1, 14.3, and 21.4 µmol of **4**. The process was initiated with approximately 1.1 GBq of azeotropically dried [^18^F]TBAF, which was resolubilized in 1200 µL of DMI. To this, 41.2 µmol of [Cu(OTf)_2_(Py)_4_] dissolved in 1200 µL of DMI was added, and the mixture was stirred at r.t. for 2 min. The resulting solution was then evenly distributed into four 600 µL portions, each containing an activity of (263–268 MBq, and transferred to separate reaction vials. Subsequently, precursor **4**, in amounts of 3.6, 7.1, 14.3, and 21.4 µmol, was introduced into the respective vials, each dissolved in 300 µL of DMI. The reaction mixtures were stirred at 110 °C for 10 min for radiofluorination. Aliquots from each vial were withdrawn and analyzed for **HSP-2** content, employing a methodology consistent with prior descriptions.

#### Amount of base (K_2_CO_3_)

To evaluate the efficacy of K_2_CO_3_ as an eluting base for [^1^⁸F]fluoride release from a QMA cartridge, the trapped [^1^⁸F]fluoride (600–800 MBq) was eluted using various mixtures: 800 µL of MeCN, 100 µL of 0.075 M TBAHCO_3_, and 100 µL of H_2_O, combined with either 0 µL, 15 µL, 100 µL, or 200 µL of an aqueous K_2_CO_3_ solution (20 mg/mL). The elution efficiency of each mixture was quantified as the ratio of released [^1^⁸F]fluoride activity to the initially trapped activity (Herth et al. 2021). Following elution, the aqueous [^1^⁸F]fluoride was azeotropically dried, and the synthesis of **[**^**1**^**⁸F]2** proceeded according to general procedure II. Each reaction commenced with approximately 100–250 MBq of azeotropically dried [^1^⁸F]fluoride, which was resolubilized in 300 µL of DMI. To this solution, 10.3 µmol of [Cu(OTf)_2_(Py)_4_], dissolved in 300 µL of DMI, was added, and the mixture was stirred at r.t. for 2 min. Subsequently, 7.1 µmol of precursor **4** in 300 µL of DMI was added. The reaction mixtures were heated at 110 °C with stirring for 10 min. The formation of **HSP-2** was monitored by HPLC analysis of aliquots withdrawn from the reaction mixtures, as detailed above.

#### Acidic protons of precursor

The impact of acidic protons such as NH in the precursor was investigated by the deuteration of **7**. Precursor **7** (25 mg) was stirred with MeOD (d_*4*_) (2 × 5 mL) for two hours at r.t. followed by solvent evaporation, followed by a third stirring with MeOD (d_*4*_) overnight to obtain deuterated **7**-*d*_*2*_. The exchange of NH with ND in precursor **7** was confirmed via NMR analysis. The effect of acidic protons on HSP formation was studied via two parallel CMRFs either **7** or **7**-*d*_*2*_, keeping the rest of the reaction conditions the same as mentioned in the general procedure II. Briefly, the azeotropically dried [^18^F]TBAF (~ 750 MBq) was dissolved in 600 µL of DMI and then 30 µmol of [Cu(OTf)_2_(Py)_4_] in 600 µL of DMI was added and stirred at r.t. for 2 min. Subsequently, the mixture was further equally divided into two reaction vials (600 µL each, corresponding to an activity of 350 MBq per portion). Afterwards, 3.4 µmol of either **7** or **7**-*d*_*2*_, dissolved in 300 µL of DMI, was added to each vial. Both reactions were then stirred at 110 °C for 10 min. After the reaction, the aliquots were taken to investigate the **HSP-1** in each reaction in a similar manner as discussed above. The reaction mixtures were quenched with the 3 mL H_2_O. Each quenched reaction mixture was loaded on preconditioned Sep-Pak® C18 plus cartridges, eluted with 2.5 mL of MeCN and further diluted with 2.5 mL H_2_O. The final 5 mL eluent was injected into the HPLC column under the conditions mentioned in Table 2. The corresponding product (**DSP-1** or **HSP-1**) was collected and concentrated under a reduced vacuum to investigate the deuteration level with HRMS. This experiment was done in duplicate (n = 2).

#### Reaction temperature

The general procedure II for the radiosynthesis of **[**^**18**^**F]2** with variations in the temperature (90, 110 and 130 °C) was used. Briefly, the azeotropically dried [^18^F]TBAF (~ 800 MBq) was dissolved in 900 µL of DMI and then 30.9 µmol of [Cu(OTf)_2_(Py)_4_] in 900 µL of DMI was added and stirred at r.t. for 2 min. Afterwards, 21.3 µmol of **4** was added in 900 µL of DMI to the mixture. The resulting reaction mixture was further divided into three reaction vials, 900 µL each (each containing activity 240–245 MBq); one reaction mixture was stirred at 90 °C; the second reaction mixture was stirred at 110 °C and the third reaction mixture at 130 °C for 10 min each. After the reaction, aliquots were taken to investigate the **HSP-2** formation in each reaction.

#### Amount of copper-complex

The radiosynthesis of **[**^**18**^**F]2** was performed following general procedure II, with modifications to investigate the effect of varying amounts of the copper-complex [Cu(OTf)_2_(Py)_4_] on the reaction outcome. Three CMRF reactions were conducted concurrently, utilizing 5.9, 10.3, and 20.7 µmol of [Cu(OTf)_2_(Py)_4_], respectively. Approximately 600 MBq of azeotropically dried [^18^F]TBAF was resolubilized in 900 µL of DMI. This solution was then equally divided into three portions (300 µL each, corresponding to an activity of 175–180 MBq per portion) and transferred into separate reaction vials. Each vial contained a pre-dissolved amount of [Cu(OTf)_2_(Py)_4_] (5.9, 10.3, or 20.7 µmol) in 300 µL of DMI. The reaction mixtures were stirred at r.t. for 2 min. Subsequently, 7.1 µmol of precursor **4**, dissolved in 300 µL of DMI, was added to each vial. The reactions were stirred at 110 °C for 10 min to facilitate the radiofluorination process. Upon completion, aliquots were withdrawn from each reaction mixture, diluted, and analyzed to assess the formation of **HSP-2**. This experimental design allowed for a systematic evaluation of the influence of [Cu(OTf)_2_(Py)_4_] concentration on the efficiency and yield of **[**^**18**^**F]2** synthesis under identical reaction conditions.

#### Comparison of different protocols A, B, C and D

The **HSP-2** formation using **[**^**18**^**F]2** with precursors **4**, **9**, or **11** was evaluated according to independent CMRFs, as summarized in Table 1. Protocols A and C: The results for Protocols A and C were obtained from experiments described in the section *“Different Leaving Groups of Precursor.”* Accordingly, the corresponding experimental procedures are detailed in that section. Protocol B: This protocol was identical to C except for the solvent system. Briefly, ~ 200 MBq of azeotropically dried [^18^F]TBAF was resolubilized in 300 µL of DMI, combined with 10.3 µmol of [Cu(OTf)_2_(Py)_4_] in 600 µL of DMI, and stirred at r.t. for 2 min. Afterwards, 7.1 µmol of compound **4** in 300 µL of *n*-BuOH was introduced and the resulting mixture were stirred at 110 °C for 10 min. After the reaction, aliquots of each reaction were taken, and the HSP-2 formation was investigated analytically in each reaction. This was investigated twice (n = 2). Protocol D was optimized based on the results of other reaction parameters (see *Results* section). Briefly, 130 MBq of azeotropically dried [^18^F]TEAF was re-dissolved in 300 µL of DMI, combined with 7 µmol of [Cu(OTf)_2_(Py)_4_] in 300 µL of DMI, and stirred at room temperature for 2 min. Subsequently, 5 µmol of precursor **9** in 300 µL of DMI was added, and the resulting mixture was stirred at 90 °C for 2 min. This experiment was performed only once, and further repetitions are required to confirm reproducibility.

## Supplementary Information

Additional file 1.

## Data Availability

The datasets generated and/or analyzed during the current study are available from the corresponding authors on reasonable request.

## Abbreviations

CMRF: Copper-mediated radiofluorination
DMI: 1,3-Dimethyl-2-imidazolidinone
DSP: Deuterated side product
EtOAc: Ethyl acetate
HPLC: High-performance liquid chromatography
HRMS: High-resolution mass spectrometry
HSP: Hydrogenated side product
LC–MS: Liquid chromatography–mass spectrometry
PE: Petroleum ether
PET: Positron emission tomography
PTC: Phase-transfer catalyst
QMA: Quaternary methylammonium, commonly used in anion exchange cartridges
RCC: Radiochemical conversion
RCY: Radiochemical yield
SPE: Solid phase extraction
TBAF: Tetrabutylammonium fluoride
TBAHCO_3_: Tetrabutylammonium hydrogencarbonate
TBAOTf: Tetrabutylammonium trifluoromethanesulfonate
TEAF: Tetraethylammonium fluoride
TEAHCO_3_: Tetraethylammonium hydrogencarbonate
TLC: Thin-layer chromatography
