# Supplementary material for: The hydrogenation side-reaction in copper-mediated radiofluorination
Source: EJNMMI Radiopharm Chem. 2025 Sep 8;10:60. doi: 10.1186/s41181-025-00384-1 (PMC12417351; doi:10.1186/s41181-025-00384-1)
Supplement: Supplementary file 1 — Additional file 1. [file 41181_2025_384_MOESM1_ESM.docx]

Supporting Information

The hydrogenation side-reaction in copper-mediated radiofluorination

Author names

Sarandeep Kaur^1,2^, Barbara Wenzel^1^, Ramona Oehme^3^, Claudia Wiesner^3^, Klaus Kopka^1,2,4,5^, and Rareş-Petru Moldovan^1,2^

Author affiliations

^1^Helmholtz-Zentrum Dresden-Rossendorf (HZDR), Institute of Radiopharmaceutical Cancer Research, Department of Experimental Neurooncological Radiopharmacy, 04318 Leipzig, Germany.

^2^Dresden University of Technology, School of Science, Faculty of Chemistry and Food Chemistry, 01069 Dresden, Germany.

^3^Leipzig University, Faculty of Chemistry, 04103 Leipzig, Germany.

^4^German Cancer Consortium (DKTK), Partner Site Dresden, 01307 Dresden, Germany.

^5^National Center for Tumor Diseases (NCT), NCT/UCC Dresden, a partnership between DKFZ, Faculty of Medicine and Univ. Hosp. Carl Gustav Carus, TU Dresden & Helmholtz-Zentrum Dresden-Rossendorf (HZDR), 01307 Dresden, Germany.

Contents

[S1: Synthesis of precursors and reference compounds 3](#_Toc206671679)

[S2: Optimization of radiofluorination 4](#_Toc206671680)

[S3: Optimization of analytical conditions 5](#_Toc206671681)

[S4: Evaluation of -Bpin vs -SnBu_3_-based precursors 9](#_Toc206671682)

[S5: Literature reported mechanisms of HSP formation 10](#_Toc206671683)

[S6: Correlation between RCC and HSP formation 12](#_Toc206671684)

[S7: D-labelling with deuterated alcohol as co-solvent 13](#_Toc206671685)

[NMR 14](#_Toc206671686)

[References 23](#_Toc206671687)

# S1: Synthesis of precursors and reference compounds

For the tetrahydropyrazolopyridine series of compounds, the synthesis of the reference compounds **1** and **HSP-1** was performed as we previously described.^1^ The precursors **3** and **7** were synthesized according to the Scheme S1. The synthesis of compound **5** is reported in our previous studies.^1^ The derivative **5** was subjected to the S_N_2 alkylation with the 4-bromomethylphenyl Bpin and 4-bromomethylphenyl iodide in the presence of base Cs_2_CO_3_ to afford compounds **3** and **6,** respectively. The iodo derivative **6** was treated with the Sn_2_Bu_6_ and Pd_2_(dba)_3_ in the presence of *i*-Pr_2_NEt in isopropanol to get stannane precursor **7**. The protons of –NH groups of **7** were deuterated by repetitive treatment with CD_3_OD to afford **7**-*d_2_*.

|  |
| --- |
| Scheme S1: Synthesis of precursors **3** and **7**. Reagents and conditions: a) ArBr, Cs_2_CO_3_, THF, r.t., 15 h [yield 48% (**3**) 78% (**6**)]; b) Sn_2_Bu_6,_ Pd_2_(dba)_3_, i-Pr_2_NEt, i-PrOH, r.t., 15 h (yield 75%); c) CD_3_OD. |

Among biphenyl-based derivatives, compound **2**, **HSP-2** and the precursors **4** and **11** were commercially available. To synthesize the biphenyl stannane precursor **8** from its iodinated analog **10**, two reaction conditions (a, b Scheme S2) were investigated. The treatment of derivative **10** with Sn_2_Bu_6_ and Pd_2_(dba)_3_ in isopropanol did not yield the desired precursor **8**. On the contrary, it resulted in the formation of biphenyl compound (**HSP-2**). Alternatively, in an attempt to synthesize **8** without degradation to biphenyl, **10** was treated with *n*-BuLi and Bu_3_SnCl in anhydrous THF under-maintained dry conditions to afford **8** (Scheme S2, I). The BEpin precursor **9** was synthesized by treating 4-biphenyl boronic acid **11** with 3,4-diethylhexane-3,4-diol in anhydrous CH_2_Cl_2_ at r.t. for 4 h (Scheme S2, II).^2^

|  |
| --- |
| Scheme S2: Synthesis of precursors **8** and **9**. Reagents and conditions: **I.** a) Sn_2_Bu_6,_ Pd_2_(dba)_3_, i-Pr_2_NEt, i-PrOH, r.t, 15 h (not working); b) n-BuLi, Bu_3_SnCl, THF, −78 °C to r.t, 1.5 h (yield 69%); **II.** c) 3,4-diethylhexane-3,4-diol, CH_2_Cl_2_, r.t, 4 h. (yield 87%). |

# S2: Optimization of radiofluorination

The CMRF reactions performed in this study are based on the classical reaction conditions used in the literature.^3–10^ The optimization of the radiolabeling procedure for the CMRF of **[^18^F]1** is reported in our previous work.^1^ Briefly, the anhydrous [^18^F]TBAF in 300 µL of DMI was stirred with 15 µmol of [Cu(OTf)₂(Py)₄] in 300 µL of DMI at r.t. for 2 min, then 3.4 µmol of **3** in 300 µL of *n*-BuOH was added and stirred at 110 °C for 10 min.^1^ The radiolabeling procedure for CMRF of **[^18^F]2** was slightly modified from the above-mentioned procedure for **[^18^F]1**. Generally, the azeotropically dried [^18^F]TBAF was dissolved in 300 µL of DMI, followed by the addition of 10.3 µmol of [Cu(OTf)₂(Py)₄] in 300 µL of DMI, stirred for 2 min at r.t., then the 7.1 µmol of precursor **4** in 300 µL of *n-*BuOH was added and the resulting reaction mixture was stirred at 110 °C for 10 min. The reaction mixture was cooled to r.t.. The identity of the radioactive products and the formation of **HSP-1** and **HSP-2** was confirmed by HPLC by spiking with reference compounds.

|  |
| --- |
| Scheme S3: CMRF of **[^18^F]1**/**[^18^F]2** with the formation of respective protodeboronated side product (**HSP-1/HSP-2**). |

# S3: Optimization of analytical conditions

Various analytical HPLC stationary and mobile phases were tested for the separation of the hydrogenated side products (**HSP-1** and **HSP-2**) from their respective (radio)fluorinated products **1** and **2**, respectively. (

Table S1). Among the evaluated stationary phases, the pentafluorophenyl propyl (PFP) column (Nucleodur PFP) with 50 or 52% MeCN and 20 mM NH₄OAc_aq_ provided efficient separation. Additionally, mobile phases containing THF with 20 mM NH₄OAc_aq_, as well as a mixture of THF/MeCN 1/1 (v/v) with 20 mM NH₄OAc_aq_, achieved separation on other stationary phases, including Reprosil-Pur C18-AQ, Reprosil-Gold C18, and Reprosil-Pur CN. However, the PFP-modified column consistently outperformed others, demonstrating superior resolution in separating fluorinated products from their respective HSPs under optimized conditions.

Table S1: Analytical investigation of a mixture of **HSP-1/HSP-2** and **1**/**2** with different reverse-phase HPLC columns and mobile phases to determine the most suitable separating conditions.

| **Column** | **Stationary phase type** | **Mobile phase (isocratic mode)** |
| --- | --- | --- |
| Reprosil-Pur 120 C18-AQ^a^ | hydrophobic, polar | -MeCN/20 mM NH_4_OAc_aq_; 1 mL/min  -MeCN/THF/20 mM NH_4_OAc_aq_; 1 mL/min |
| Reprosil-Gold 120 C18^a^ | strong hydrophobic | -MeCN/20 mM NH_4_OAc_aq_; 1 mL/min  -MeCN/THF/20 mM NH_4_OAc_aq_; 1 mL/min  -THF/20 mM NH_4_OAc_aq_ ; 0.7 mL/min |
| Reprosil-Pur Basic-C18-HD^a^ | strong hydrophobic | -MeCN/20 mM NH_4_OAc_aq_; 1 mL/min |
| Reprosil-Pur 120 Phenyl^a^ | hydrophobic, polar | -MeCN/20 mM NH_4_OAc_aq_; 1 mL/min  -MeOH/20 mM NH_4_OAc_aq_; 0.7 mL/min  -MeOH/0.05% TFA_aq_; 0.7 mL/min |
| Reprosil-Pur 120 CN^a^ | hydrophobic, polar | -MeCN/20 mM NH_4_OAc_aq_; 1 mL/min  -MeCN/0.05% TFA_aq_; 1 mL/min  -MeOH/20 mM NH_4_OAc_aq_; 0.7 mL/min  -MeOH/0.05% TFA_aq_; 0.7 mL/min  -MeCN/THF/20 mM NH_4_OAc_aq_; 1 mL/min  -THF/20 mM NH_4_OAc_aq_; 0.7 mL/min |
| Nucleoshell^b^ | hydrophobic | -MeCN/20 mM NH_4_OAc_aq_; 0.4 mL/min |
| X Bridge^c^ | n/a | -MeCN/20 mM NH_4_OAc_aq_; 0.4 mL/min |
| Nucleodur sphrinx^a^ | hydrophobic, polar | -MeCN/20 mM NH_4_OAc_aq_; 1 mL/min |
| Nucleodur PFP^a^ | hydrophobic, polar, steric selectivity | -MeCN/20 mM NH_4_OAc_aq_; 1 mL/min |
| ^a^250*4.6 mm, 5 µm particle size  ^b^150*3.0 mm, 3.5 µm particle size  ^c^150*3.0 mm, 3.5 µm particle size | | |

|    |
| --- |
| Figure S1: Representative chromatograms for the separation of of **HSP-1**/**HSP-2** and **1**/**2,**^a^250*4.6 mm, 5 µm particle size; ^b^flow 1 mL/min |

**Calibration curves of HSP-1/HSP-2**

The amounts of **HSP-1**/**HSP-2** were determined by analytical HPLC. The **HSP-1** and **HSP-2** were calibrated with their corresponding reference compounds. The different concentrations (1-100 µg/mL) of the reference were prepared in MeCN/H_2_O (1/1, *v/v*). Then, 50 µL of each solution was applied directly onto the selected HPLC column and UV detection was carried out at the absorption maximum of the corresponding radiotracer (268 nm for **HSP-1** and 254 nm for **HSP-2**). The resulting peak area (mV min) was plotted with the corresponding masses in the different concentrations ( and ). After each radiosynthesis, 30 µL of the reaction mixture was diluted with MeCN/H_2_O (1/1, *v/v*) to a final volume of 100 µL and then injected into the HPLC. The amount of the **HSP-1/HSP-2** compound was calculated by the resulting peak area and the linear regression of the calibration curve.


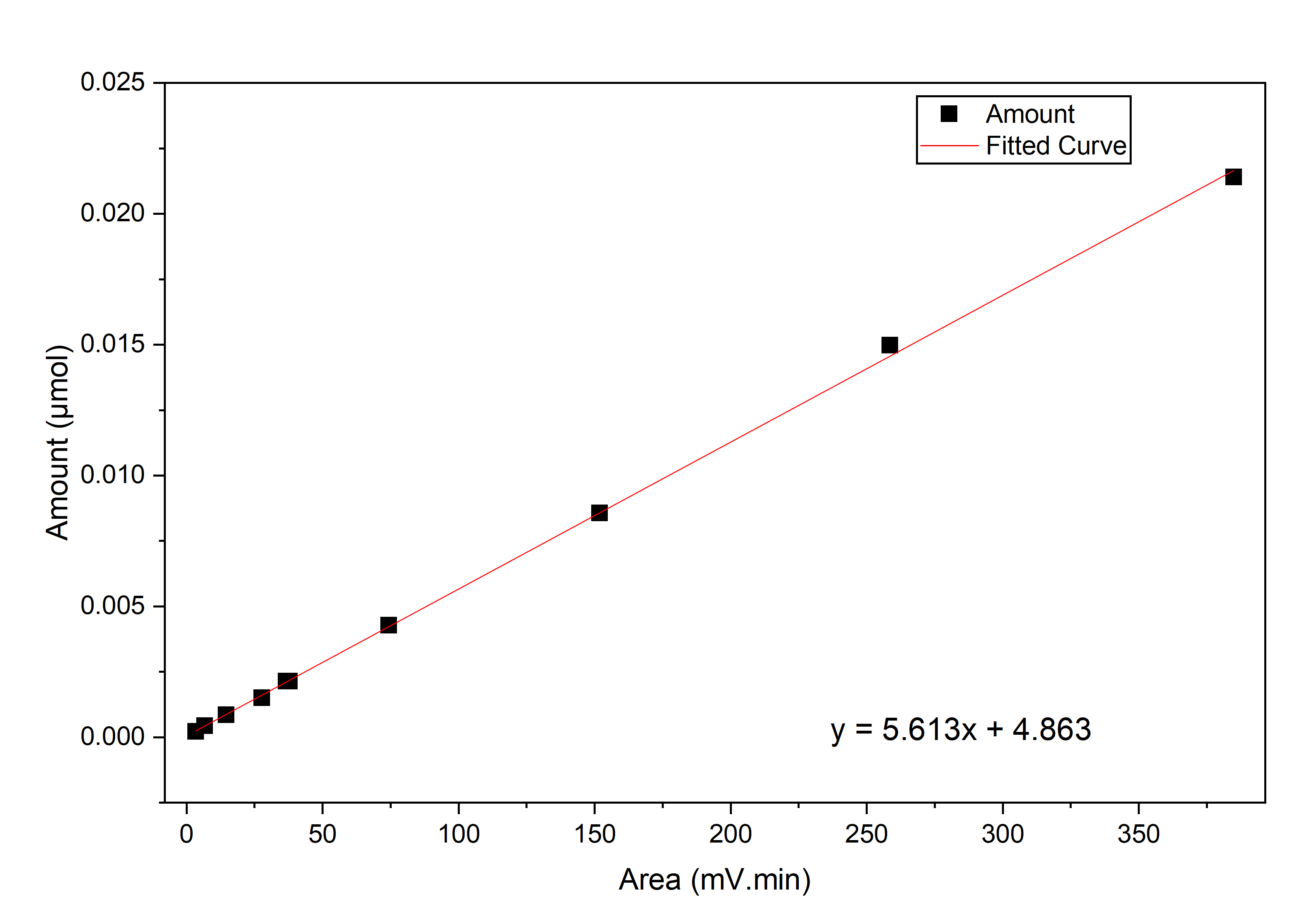


Figure S2: Calibration curve of HSP-1; Nucleodur PFP, 250 x 4.6 mm, 5 µm and 52% MeCN, 20 mM NH_4_OAc_aq_, 1 mL/min, 268 nm.


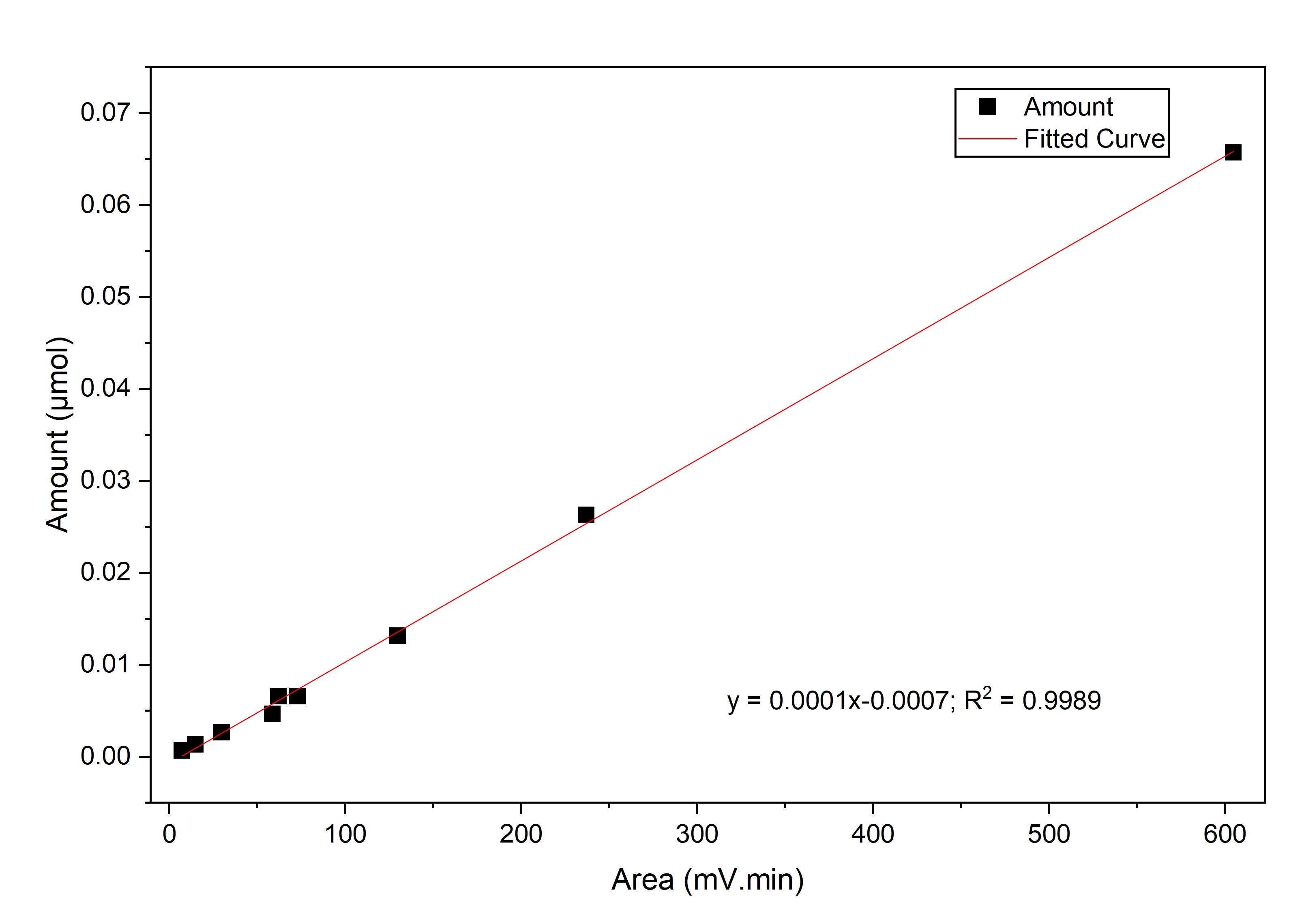


Figure S3: Calibration curve of **HSP-2**; Nucleodur PFP, 250 x 4.6 mm, 5 µm and 50% MeCN, 20 mM NH_4_OAc_aq_, 1 mL/min, 254 nm.

# S4: Evaluation of -Bpin vs -SnBu_3_-based precursors

|  |  |
| --- | --- |
| Figure S4: Dependency of **HSP-1** formation and RCC on precursor type for CMRF of **[^18^F]1**. | Figure S5: Dependency of **HSP-2** formation and RCC on precursor type for CMRF of **[^18^F]2**. |
|  | |
| Figure S6: HPLC chromatograms of the precursor **8**. Solutions preparations: 30 µL of 1 mg/mL of **8** was diluted to 100 µL either with MeCN or MeCN/H_2_O 1/1 (v/v) and analyzed with HPLC at different time points. HPLC conditions: Reprosil-Pur C18-AQ, 66% MeCN/20 mM NH_4_OAc_aq_; 1 mL/min. | |

# S5: Literature reported mechanisms of HSP formation

| **** |
| --- |
| Scheme S4: General possible mechanism proposed by Nolan et al. 2004, for the HSP formation in Pd-catalyzed coupling reactions in organic chemistry.^11^ |
| **** |
| Scheme S5: General mechanism proposed by Ahmadi et al.2013, showing the role of alcohol in the HSP formation in Pd-catalysed coupling reactions in organic chemistry.^12^ |
| **** |
| Scheme S6: Auto/self-condensation property of the boronic acids to yield boroxines and water.^13^ |
| **** |
| Scheme S7: Stepwise (IA) and Concerted (IB) Mechanisms for base-catalyzed arylboronic acid protodeboronation (HSP formation) reported by Cox et al. 2017.^14^ |

# S6: Correlation between RCC and HSP formation

|  |  |
| --- | --- |
|  |  |
|  |  |
|  |  |
| Figure S7: Correlation between RCC of **[^18^F]1** and **[^18^F]2** versus the formation of **HSP-1** and **HSP-2** under various CMRF reaction parameters. **A**) the correlation between RCC and HSP formation when DMI:n-BuOH (2:1) is used as solvent system in comparison to DMI alone; **B**) the correlation between RCC and HSP formation with different PTCs and reaction time; **C**) the correlation between RCC and HSP formation with increasing amount of precursor; **D**) the correlation between RCC and HSP formation with various amounts of K_2_CO_3_ used as a base; **E**) & **F**) the correlation between RCC and HSP formation when different leaving groups of precursor is used; **G**) the correlation between RCC and HSP formation with varying reaction temperature; and **H**) the correlation between RCC and HSP formation with increasing amount of copper-complex ([Cu(OTf)₂(Py)₄]). | |

# S7: D-labelling with deuterated alcohol as co-solvent


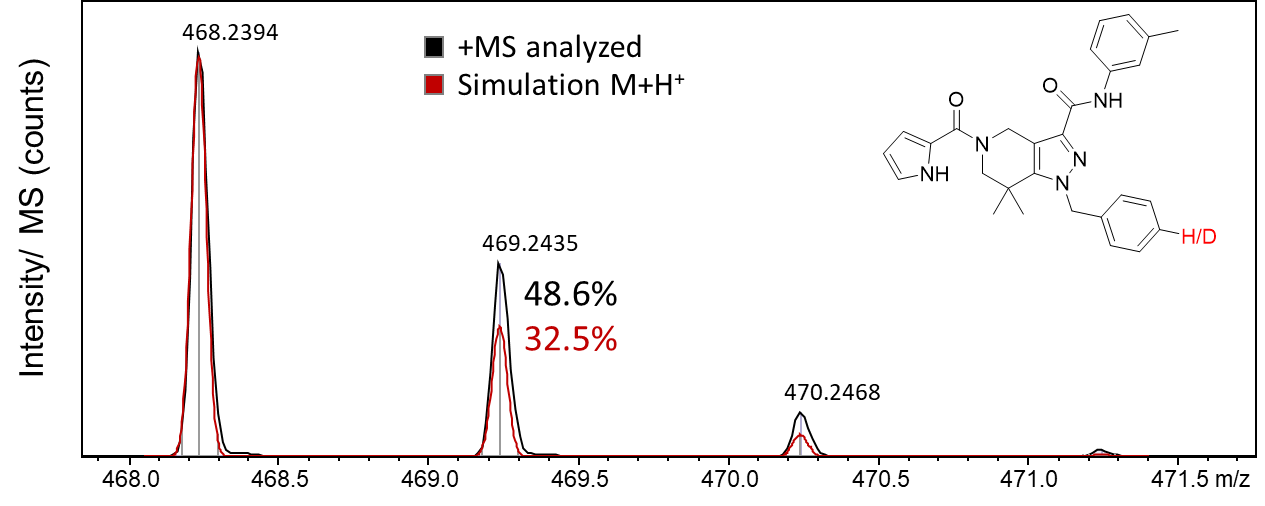


Figure S8: D-labelling with deuterated alcohol as co-solvent, overlay of the obtained mass spectrum with a simulation of protonated HSP-1 (C_28_H_29_N_5_O_2_, m/z 468.2394). The simulation of the isotopic pattern results in an intensity of 32.5% of the M+1 peak with m/z 469.2424. The higher abundant M+1 peak of the obtained spectrum with 48.6% suggests the presence of DSP-1 (C_28_H_28_DN_5_O_2_, m/z 469.2457) with a contribution of 6.1% compared to the signal intensity of HSP-1, respectively a cumulative share of 5.75%. DSP-1 features a higher mass than the M+1 of HSP-1 (by 0.0033 u). While the mass resolution is not sufficient to separate the two ionic species, the presence of DSP-1 is also indicated by a shift towards higher m/z of the composite peaks M+1/MD (m/z 469.2435 instead of m/z 469.2424 because of overlay with m/z 469.2457 of DSP-1) and M+2/MD+1 (m/z 470.2468 instead of m/z 470.2453 because of overlay with m/z 470.2487, respectively). Note that analysis of the aqueous quench with D_2_O obtained the predicted m/z 469.2424 with an intensity of 32.5% corresponding to the expected exact mass and relative intensity of the M+1 isotope of HSP-1, confirming that the aqueous quenching of the reaction did not contribute to the HSP formation.

# NMR


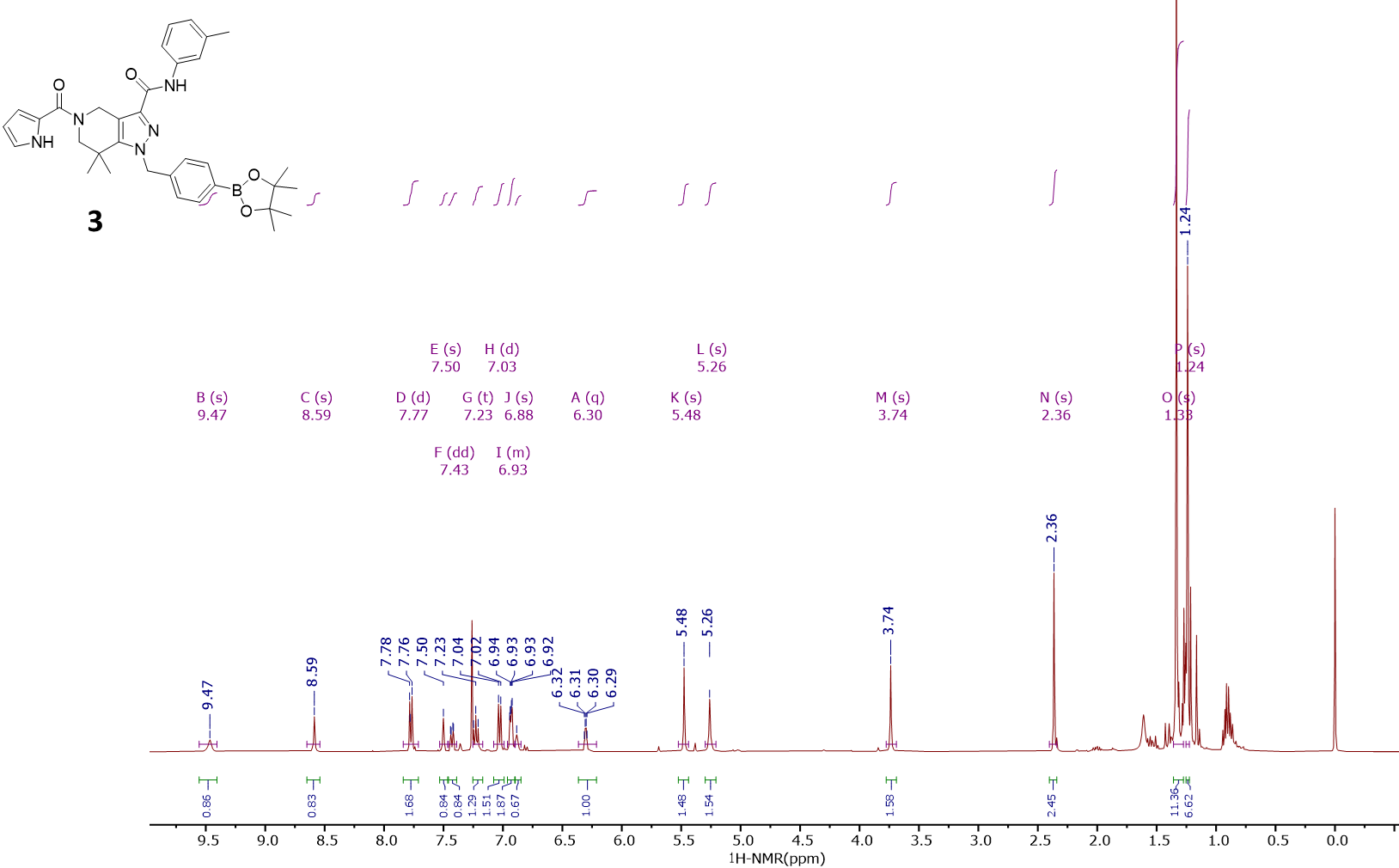


Figure S9: ^1^H-NMR of **3** in CDCl_3_


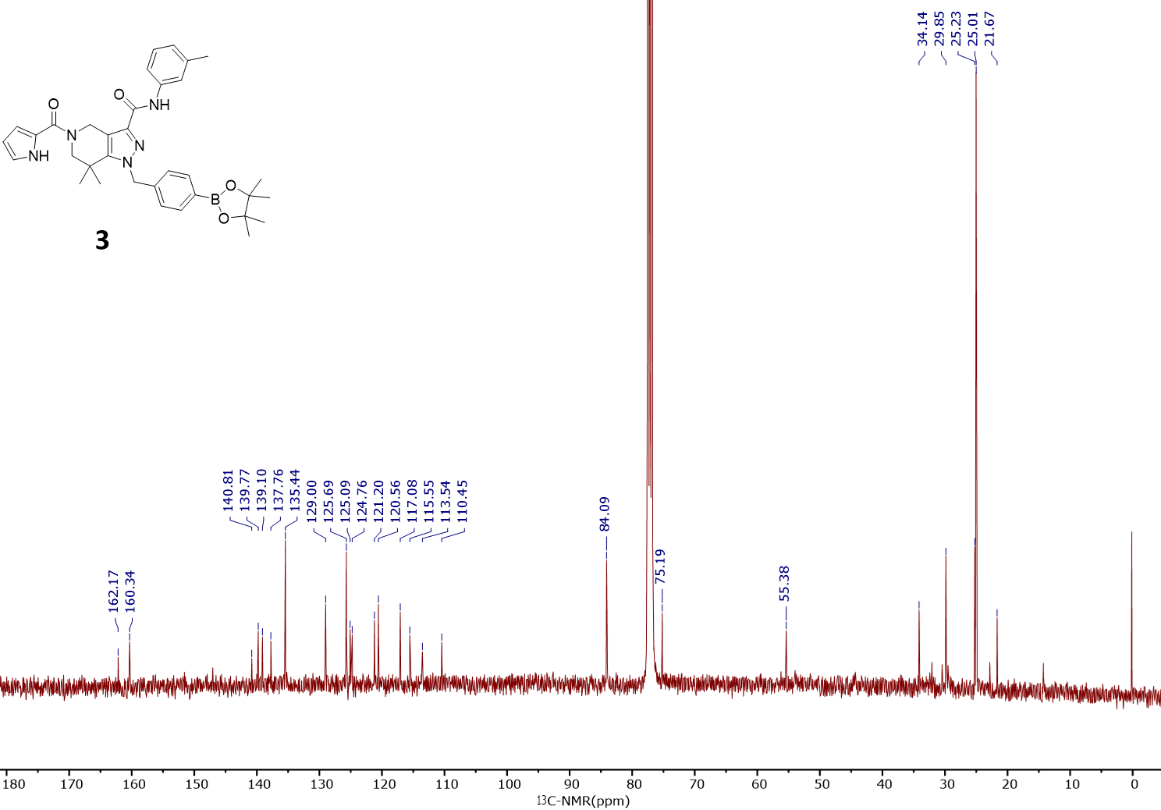


Figure S10: ^13^C-NMR of **3** in CDCl_3_


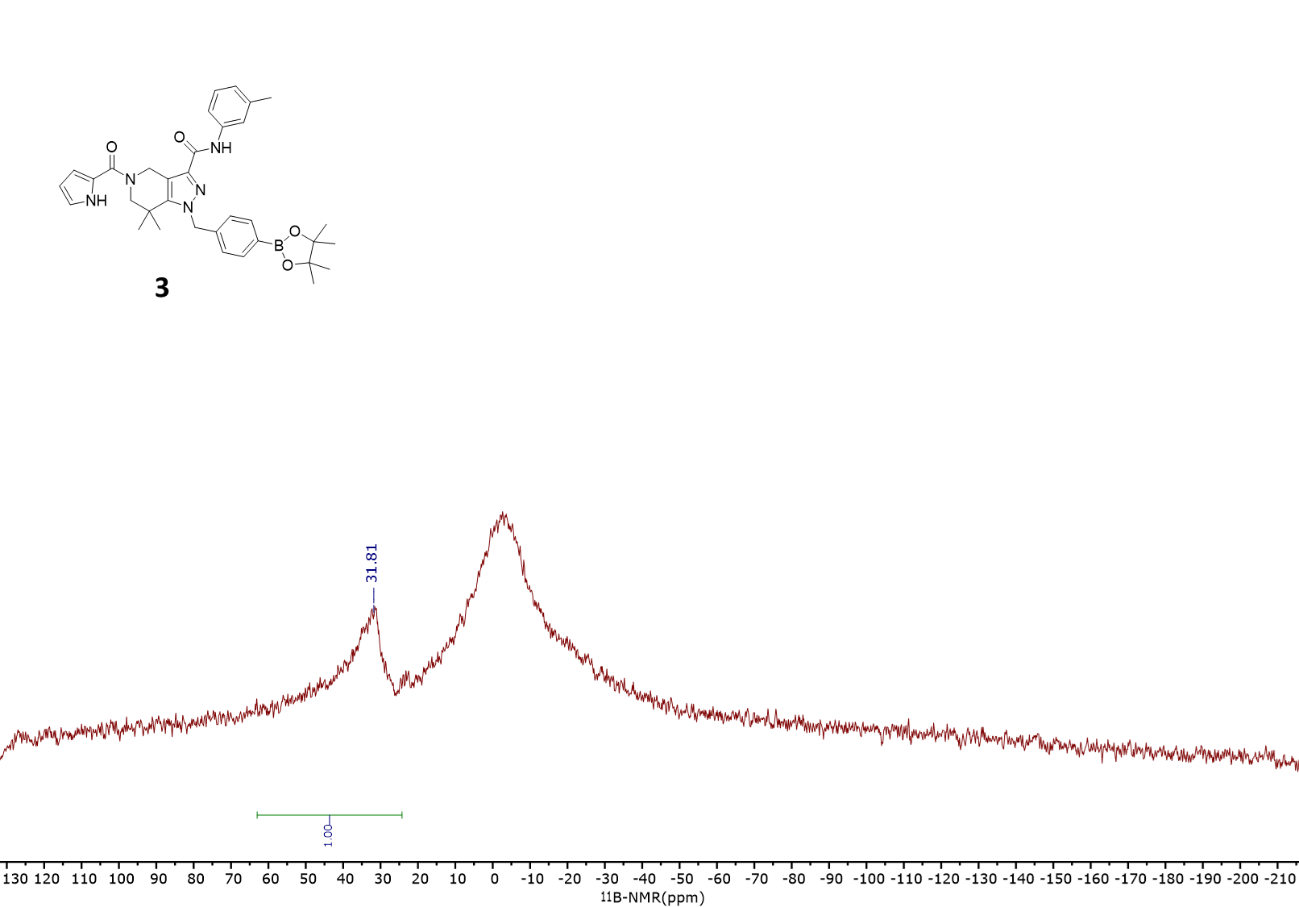


Figure S11: ^13^B NMR of **3** in CDCl_3_


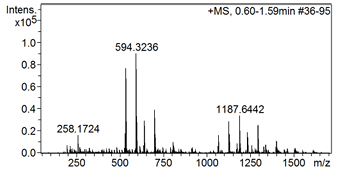


Figure S12: HRMS (ESI+): m/z (%) of **3**


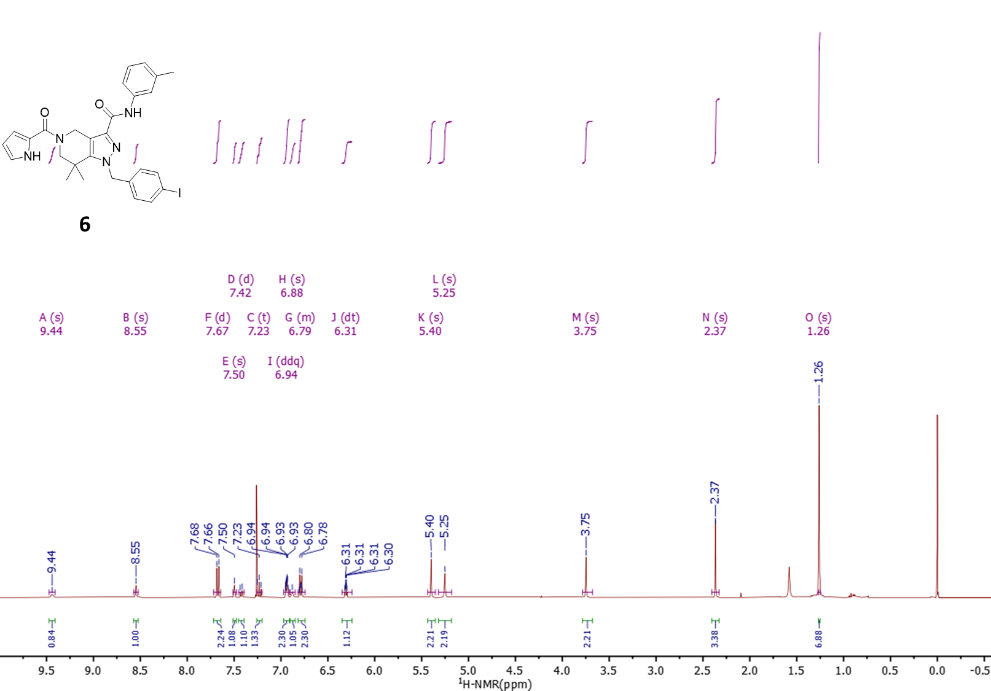


Figure S13: ^1^H-NMR of **6** in CDCl_3_


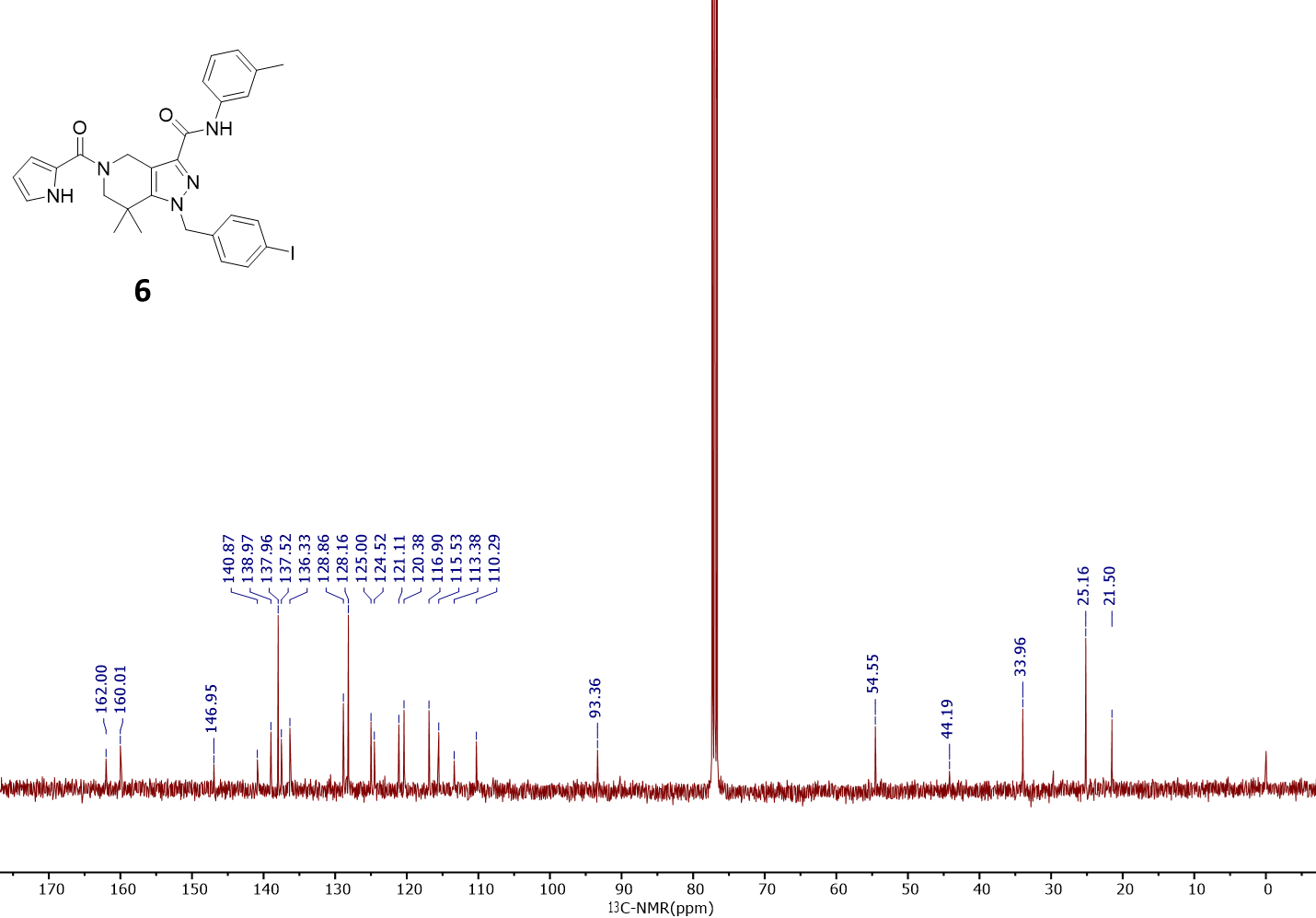


Figure S14: ^13^C-NMR of **6** in CDCl_3_


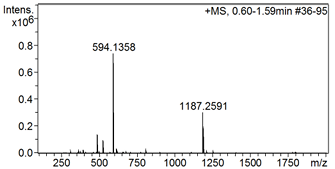


Figure S15: HRMS (ESI+): m/z (%) of **6**


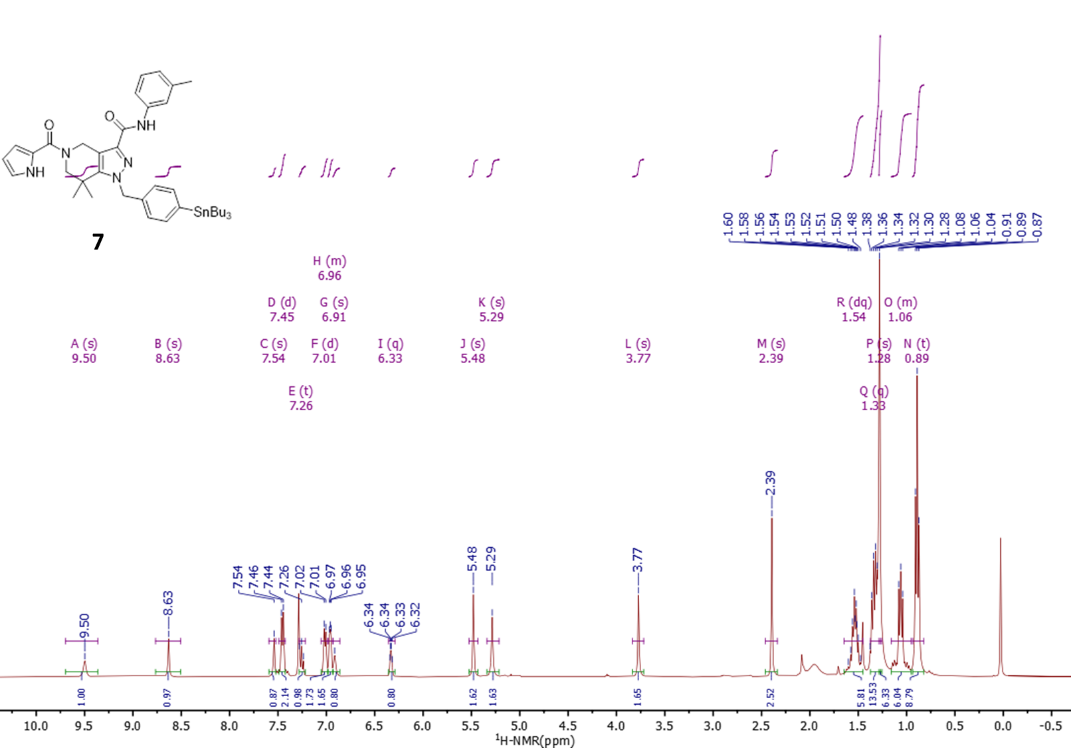


Figure S16: ^1^H-NMR of **7** in CDCl_3_


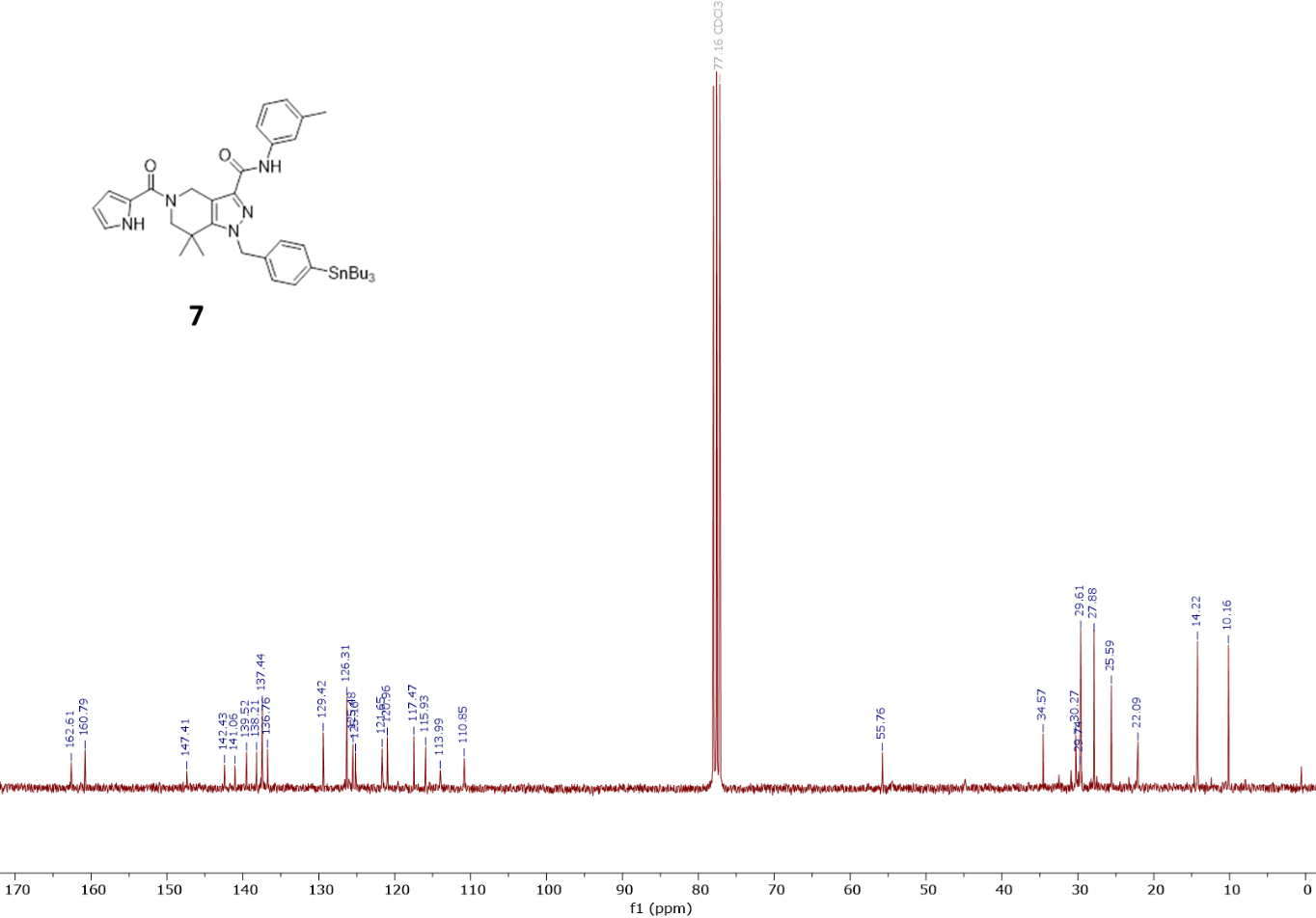


Figure S17: ^13^C-NMR of **7** in CDCl_3_


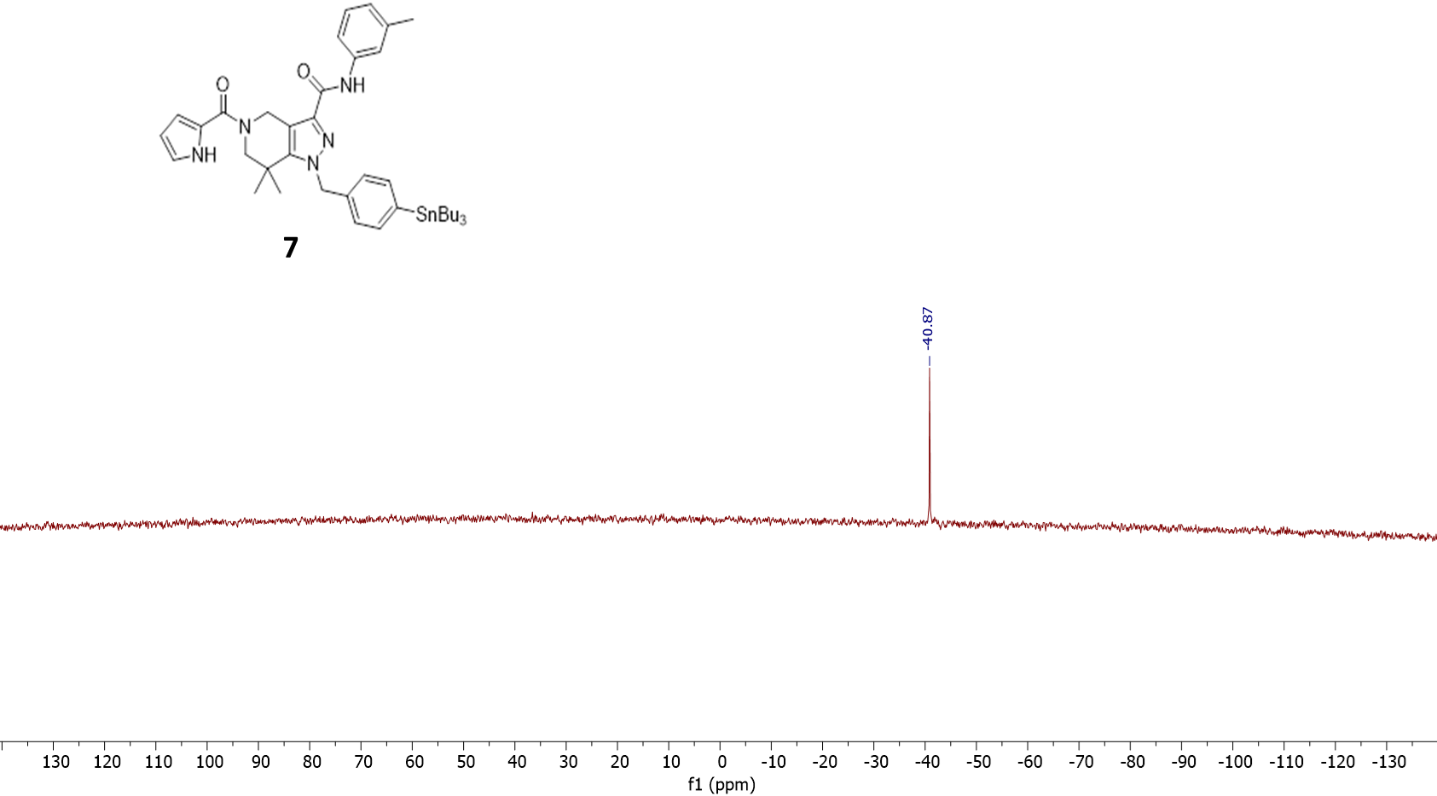


Figure S18: ^119^Sn-NMR of **7** in CDCl_3_


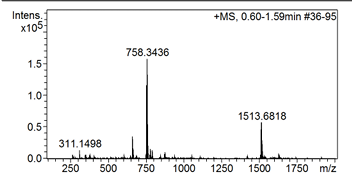


Figure S19: HRMS (ESI+): m/z (%) of **7**


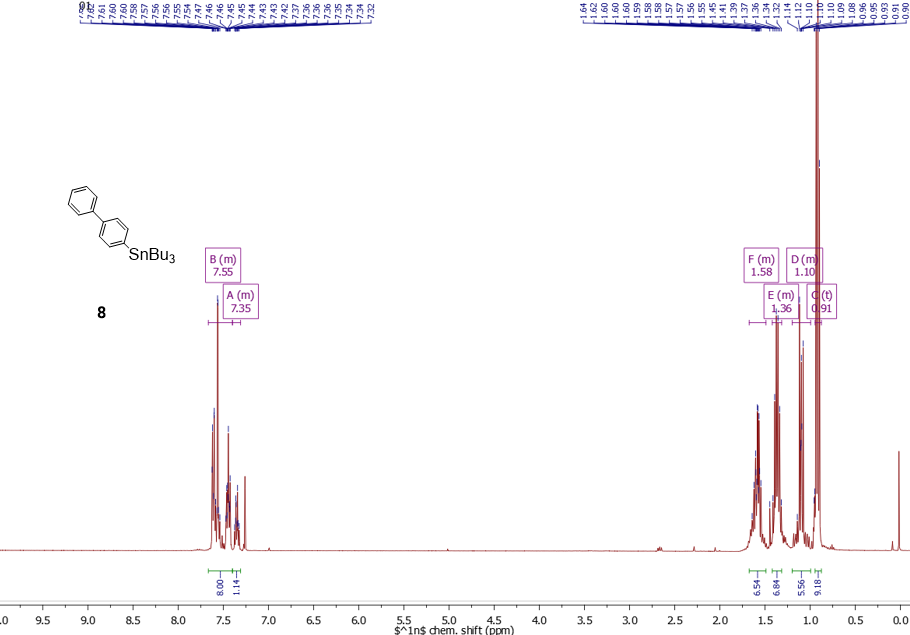


Figure S20: ^1^H-NMR of **8** in CDCl_3_


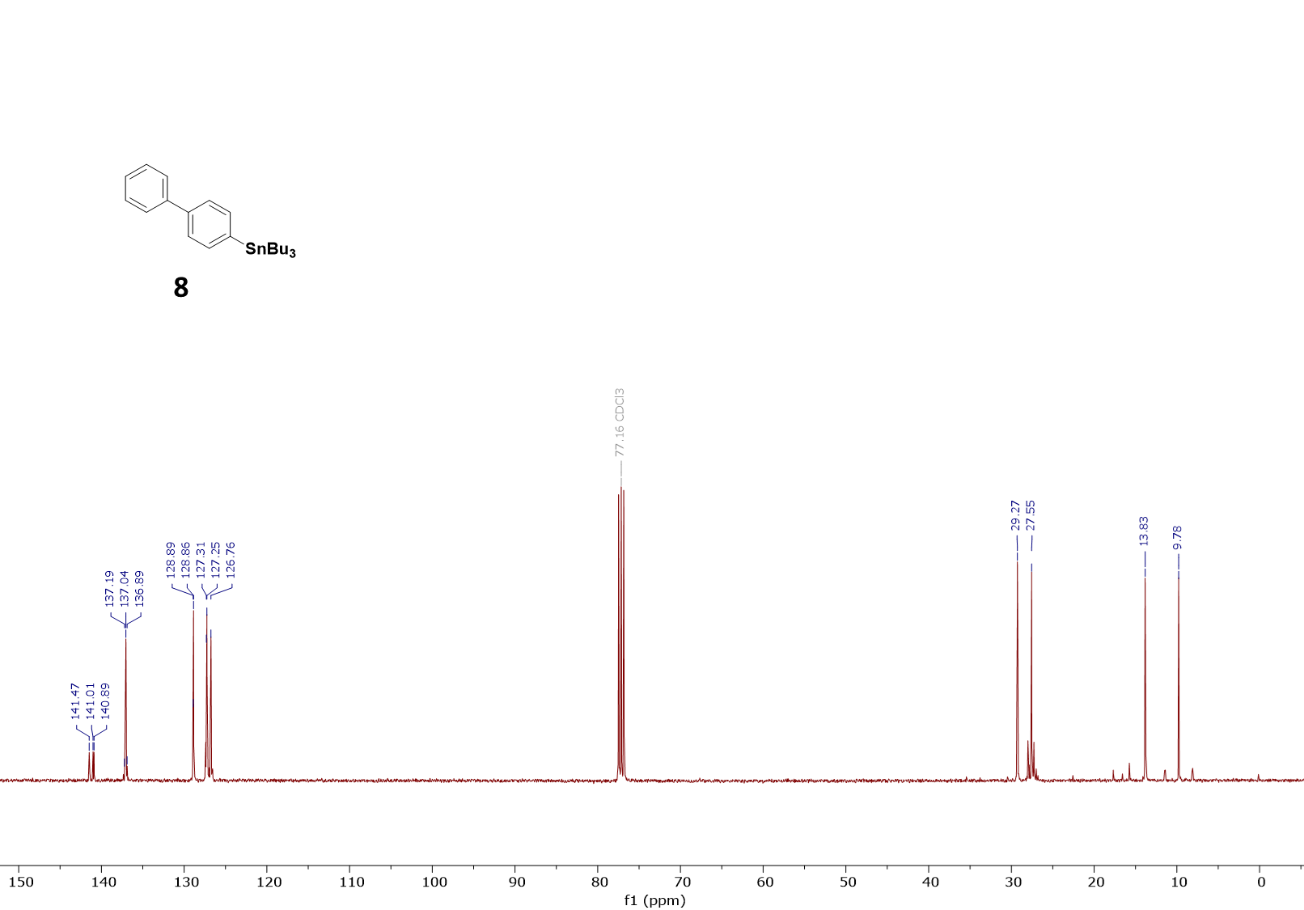


Figure S21: ^13^C-NMR of **8** in CDCl_3_


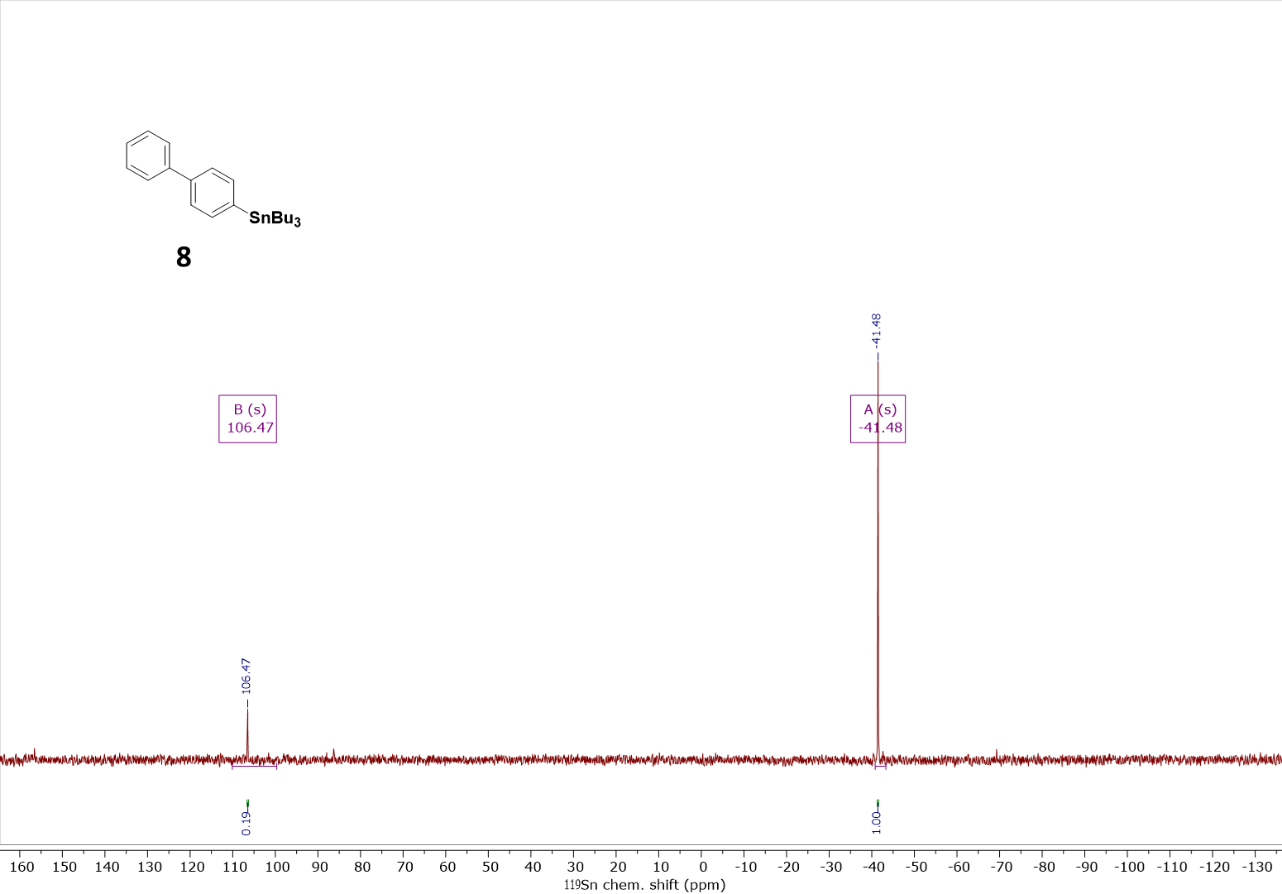


Figure S22: ^119^Sn-NMR of **8** in CDCl_3_


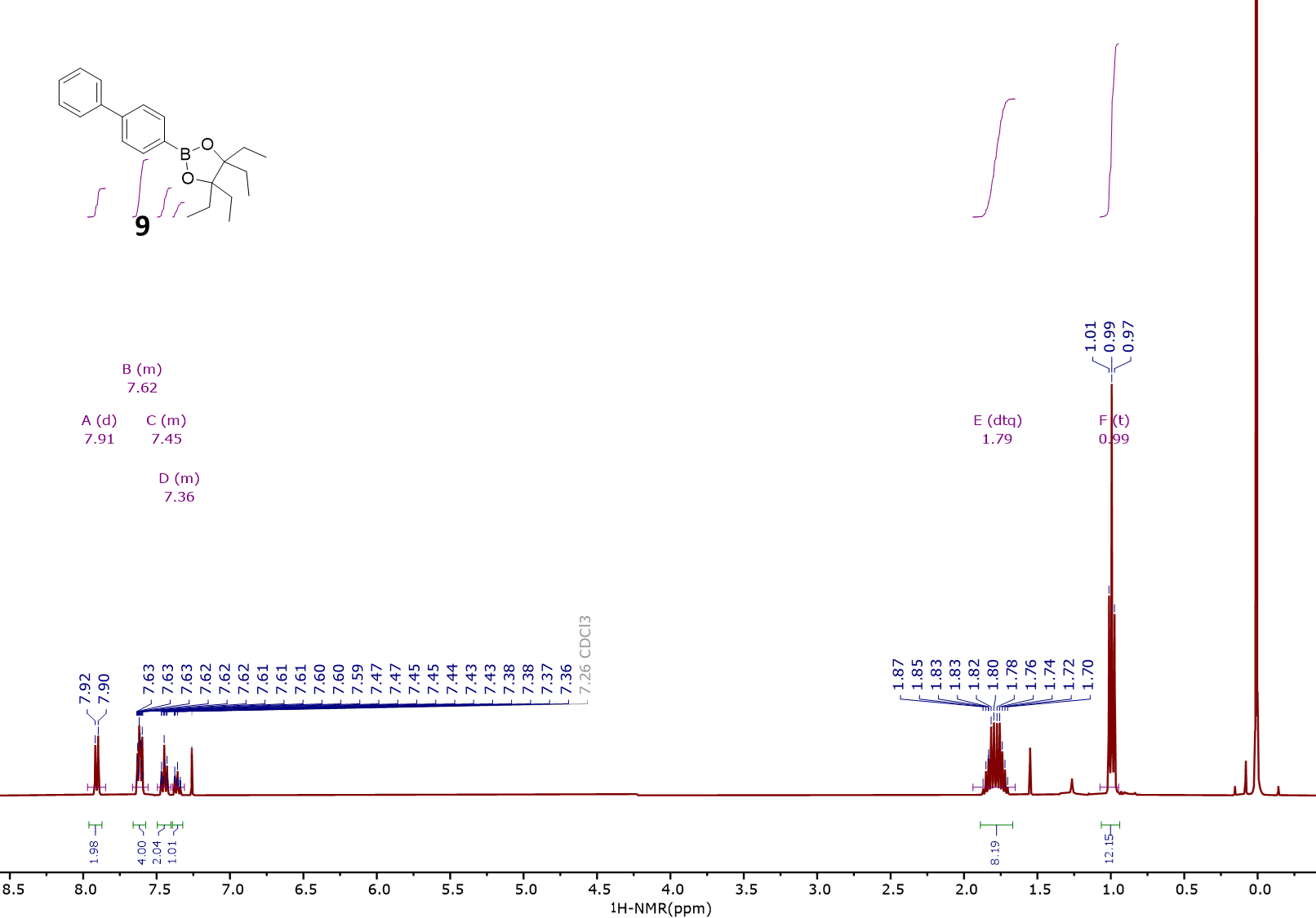


Figure S23: ^1^H-NMR of **9** in CDCl_3_


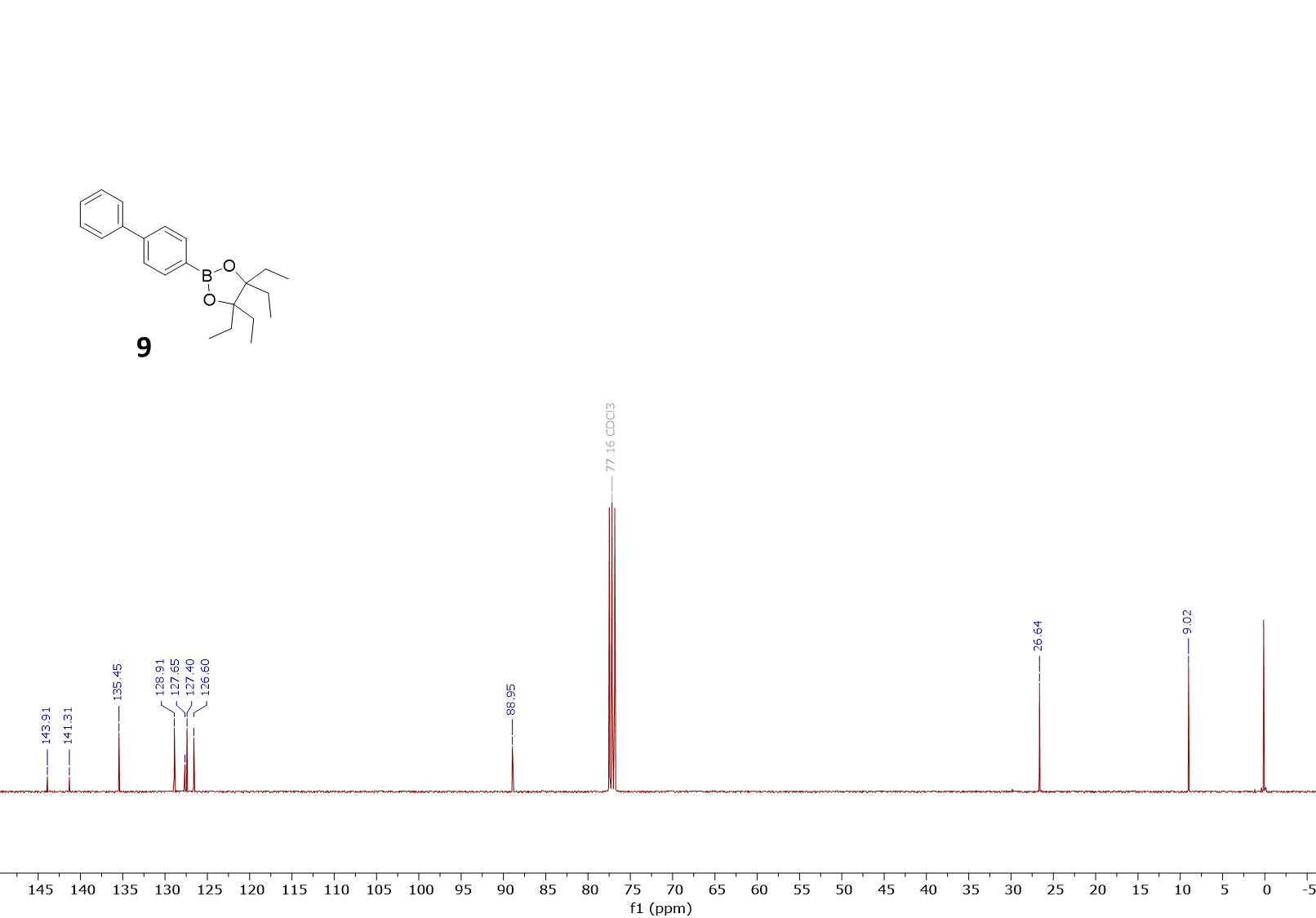


Figure S24: ^13^C-NMR of **9** in CDCl_3_


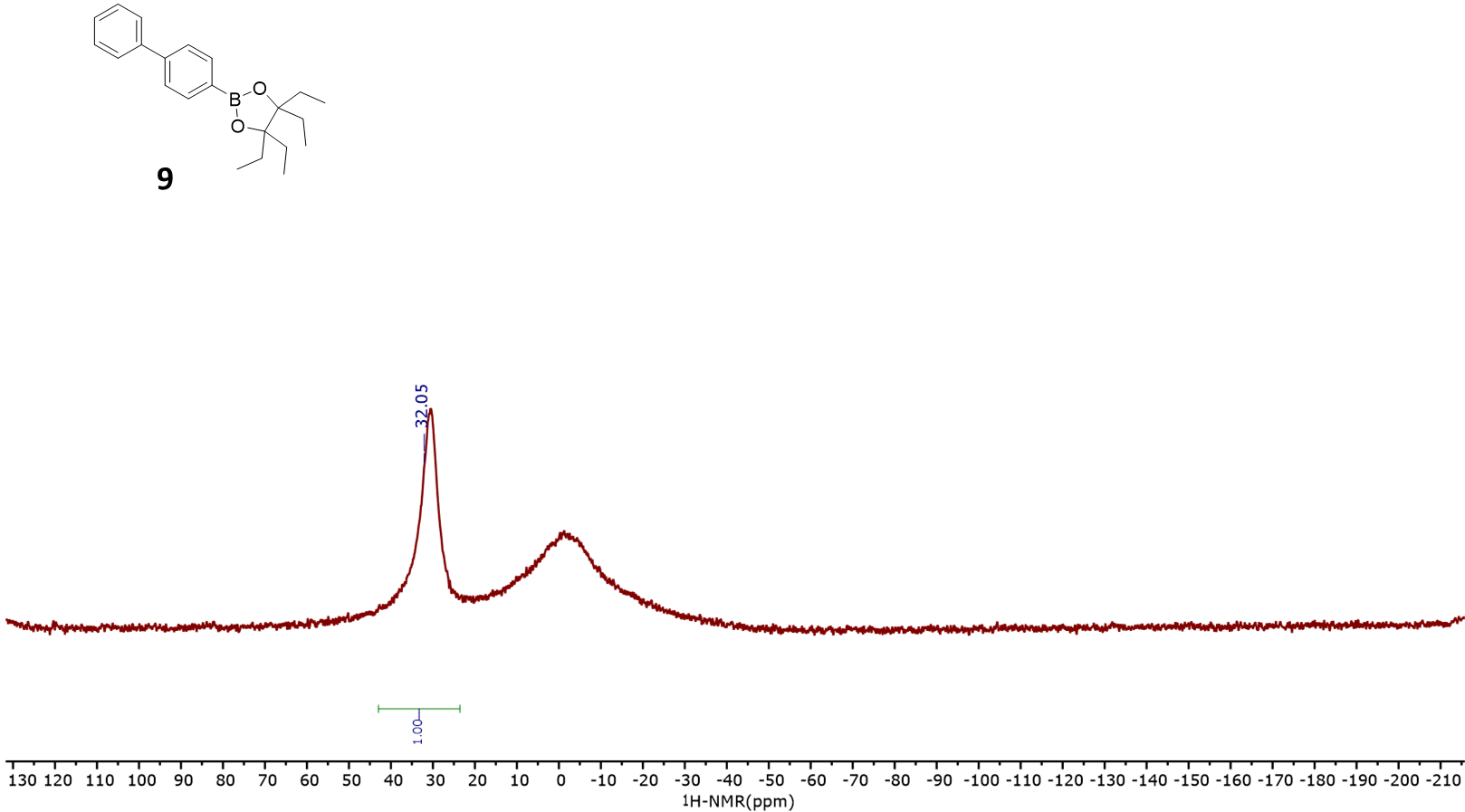


Figure S25: ^11^B-NMR of **9** in CDCl_3_

# References

(1) Kaur, S.; Dukic-Stefanovic, S.; Deuther-Conrad, W.; Toussaint, M.; Wenzel, B.; Lönnecke, P.; Donat, C. K.; Moldovan, R.-P.; Kopka, K. From Inhibitors to PET: SAR-Based Development of [18F]SK60 for mIDH1 Imaging. *J. Med. Chem.* **2025**, *68* (13), 13750–13771. https://doi.org/10.1021/acs.jmedchem.5c00584.

(2) Oka, N.; Yamada, T.; Sajiki, H.; Akai, S.; Ikawa, T. Aryl Boronic Esters Are Stable on Silica Gel and Reactive under Suzuki–Miyaura Coupling Conditions. *Org. Lett.* **2022**, *24* (19), 3510–3514. https://doi.org/10.1021/acs.orglett.2c01174.

(3) Jacobson, O.; Kiesewetter, D. O.; Chen, X. Fluorine-18 Radiochemistry, Labeling Strategies and Synthetic Routes. *Bioconjug. Chem.* **2015**, *26* (1), 1–18. https://doi.org/10.1021/bc500475e.

(4) Cai, L.; Lu, S.; Pike, V. W. Chemistry with [18F]Fluoride Ion. *Eur. J. Org. Chem.* **2008**, *2008* (17), 2853–2873. https://doi.org/10.1002/ejoc.200800114.

(5) Richarz, R.; Krapf, P.; Zarrad, F.; Urusova, E. A.; Neumaier, B.; Zlatopolskiy, B. D. Neither Azeotropic Drying, nor Base nor Other Additives: A Minimalist Approach to 18F-Labeling. *Org. Biomol. Chem.* **2014**, *12* (40), 8094–8099. https://doi.org/10.1039/C4OB01336K.

(6) Cole, L. E.; Stewart, N. M.; Littich, R.; Hoareau, R.; Scott, J. H. P. Radiosyntheses Using Fluorine-18: The Art and Science of Late Stage Fluorination. *Curr. Top. Med. Chem.* **2014**, *14* (7), 875–900. http://dx.doi.org/10.2174/1568026614666140202205035.

(7) Zlatopolskiy, B. D.; Zischler, J.; Krapf, P.; Zarrad, F.; Urusova, E. A.; Kordys, E.; Endepols, H.; Neumaier, B. Copper-Mediated Aromatic Radiofluorination Revisited: Efficient Production of PET Tracers on a Preparative Scale. *Chem. Weinh. Bergstr. Ger.* **2015**, *21* (15), 5972–5979. https://doi.org/10.1002/chem.201405586.

(8) Brooks E; Wu X; Hanel A; Nguyen S; Wang J; Zhang JH; Harrison A; Zhang W. Identification and Characterization of Small-Molecule Inhibitors of the R132H/R132H Mutant Isocitrate Dehydrogenase 1 Homodimer and R132H/Wild-Type Heterodimer. *J. Biomol. Screen.* **2014**, *19* (8). https://doi.org/10.1177/1087057114541148.

(9) Tredwell, M.; Preshlock, S. M.; Taylor, N. J.; Gruber, S.; Huiban, M.; Passchier, J.; Mercier, J.; Génicot, C.; Gouverneur, V. A General Copper-Mediated Nucleophilic 18F Fluorination of Arenes. *Angew. Chem. Int. Ed.* **2014**, *53* (30), 7751–7755. https://doi.org/10.1002/anie.201404436.

(10) Wright, J. S.; Kaur, T.; Preshlock, S.; Tanzey, S. S.; Winton, W. P.; Sharninghausen, L. S.; Wiesner, N.; Brooks, A. F.; Sanford, M. S.; Scott, P. J. H. Copper-Mediated Late-Stage Radiofluorination: Five Years of Impact on Preclinical and Clinical PET Imaging. *Clin. Transl. Imaging* **2020**, *8* (3), 167–206. https://doi.org/doi:10.1007/s40336-020-00368-y.

(11) Navarro, O.; Kaur, H.; Mahjoor, P.; Nolan, S. P. Cross-Coupling and Dehalogenation Reactions Catalyzed by (N-Heterocyclic Carbene)Pd(Allyl)Cl Complexes. *J. Org. Chem.* **2004**, *69* (9), 3173–3180. https://doi.org/10.1021/jo035834p.

(12) Ahmadi, Z.; McIndoe, J. S. A Mechanistic Investigation of Hydrodehalogenation Using ESI-MS. *Chem. Commun.* **2013**, *49* (98), 11488–11490. https://doi.org/10.1039/C3CC46271D.

(13) Noonan, G.; Leach, A. G. A Mechanistic Proposal for the Protodeboronation of Neat Boronic Acids: Boronic Acid Mediated Reaction in the Solid State. *Org. Biomol. Chem.* **2015**, *13* (9), 2555–2560. https://doi.org/10.1039/c4ob02543a.

(14) Cox, P. A.; Reid, M.; Leach, A. G.; Campbell, A. D.; King, E. J.; Lloyd-Jones, G. C. Base-Catalyzed Aryl-B(OH)2 Protodeboronation Revisited: From Concerted Proton Transfer to Liberation of a Transient Aryl Anion. *J. Am. Chem. Soc.* **2017**, *139* (37), 13156–13165. https://doi.org/10.1021/jacs.7b07444.
